# Laminin-associated integrins mediate Diffuse Intrinsic Pontine Glioma infiltration and therapy response within a neural assembloid model

**DOI:** 10.1186/s40478-024-01765-4

**Published:** 2024-05-05

**Authors:** Sauradeep Sinha, Michelle S. Huang, Georgios Mikos, Yudhishtar Bedi, Luis Soto, Sarah Lensch, Manish Ayushman, Lacramioara Bintu, Nidhi Bhutani, Sarah C. Heilshorn, Fan Yang

**Affiliations:** 1 Department of Bioengineering, Stanford University, Stanford, CA 94305 USA; 2 Department of Chemical Engineering, Stanford University, Stanford, CA 94305 USA; 3 Departments of Orthopaedic Surgery and Bioengineering, Stanford University, 240 Pasteur Dr., Biomedical Innovation Building 1254, Palo Alto, CA 94305 USA; 4 Department of Radiation Oncology, Stanford University, Stanford, CA 94305 USA; 5 Department of Materials Science and Engineering, Stanford University, 476 Lomita Mall, McCullough Building, Room 246, Palo Alto, CA 94305 USA

**Keywords:** Diffuse Intrinsic Pontine Glioma, Neural organoids, Extracellular matrix, Integrins, Laminin

## Abstract

Diffuse Intrinsic Pontine Glioma (DIPG) is a highly aggressive and fatal pediatric brain cancer. One pre-requisite for tumor cells to infiltrate is adhesion to extracellular matrix (ECM) components. However, it remains largely unknown which ECM proteins are critical in enabling DIPG adhesion and migration and which integrin receptors mediate these processes. Here, we identify laminin as a key ECM protein that supports robust DIPG cell adhesion and migration. To study DIPG infiltration, we developed a DIPG-neural assembloid model, which is composed of a DIPG spheroid fused to a human induced pluripotent stem cell-derived neural organoid. Using this assembloid model, we demonstrate that knockdown of laminin-associated integrins significantly impedes DIPG infiltration. Moreover, laminin-associated integrin knockdown improves DIPG response to radiation and HDAC inhibitor treatment within the DIPG-neural assembloids. These findings reveal the critical role of laminin-associated integrins in mediating DIPG progression and drug response. The results also provide evidence that disrupting integrin receptors may offer a novel therapeutic strategy to enhance DIPG treatment outcomes. Finally, these results establish DIPG-neural assembloid models as a powerful tool to study DIPG disease progression and enable drug discovery.

**Supplementary Information:**

The online version contains supplementary material available at 10.1186/s40478-024-01765-4.

## Introduction

Diffuse Intrinsic Pontine Glioma (DIPG) is a fatal brainstem tumor that impacts mostly children and has a median survival of less than one year [1]. DIPG is highly infiltrative and invades into anatomically distant brain regions [2, 3]. Previous studies have revealed that DIPG tumors are epigenetically dysregulated, which leads to aberrant transcription and behavior [4]. However, cell-intrinsic mechanisms are not sufficient to explain disease progression and spread. In adult brain tumors such as glioblastoma (GBM), the extracellular matrix (ECM) has been shown to play a critical role in modulating GBM behavior such as invasion and treatment resistance [5]. However, the role of ECM in driving DIPG progression remains largely unknown.

One pre-requisite for glioma cells to infiltrate throughout the brain is adhesion to the ECM [6]. Cell-ECM adhesions are governed by integrin receptors [5, 6], and integrin subunits such as αV and β1 have been implicated as contributors to GBM invasion [5]. Targeting integrin receptors with Cilengitide, an inhibitor of αvβ3 and αvβ5, has demonstrated modest anti-tumor activity in GBM patients [7]. However, previous studies on targeting integrin receptors were limited to adult brain tumors. To date, it remains largely unknown which ECM proteins and integrin receptors are critical in mediating DIPG adhesion and migration.

Another challenge for DIPG is a lack of treatment options. Radiation is the only standard-of-care therapy available for patients. Surgical resection is not possible because DIPG grows diffusely within critical brainstem structures, and chemotherapy is ineffective for DIPG [1]. While the multiple histone deacetylase inhibitor, panobinostat, has demonstrated therapeutic efficacy in orthotopic xenograft models, DIPG ultimately develops resistance—highlighting the need for combinational therapies and additional therapeutic targets [8]. To address the above unmet needs, we sought to identify key ECM proteins mediating DIPG adhesion and investigate the role of associated integrins on DIPG invasion and therapy response. To study DIPG invasion, we developed a DIPG-neural assembloid as a novel in vitro experimental model. Using this model, we demonstrate that targeting integrin receptors that mediate DIPG adhesion may offer a promising strategy to reduce DIPG infiltration and further improve DIPG therapy responses.

## Results

### Laminin and, to a lesser extent, fibronectin impact DIPG migration and response to radiation and panobinostat treatment in 2D culture

**Fig. 1:**
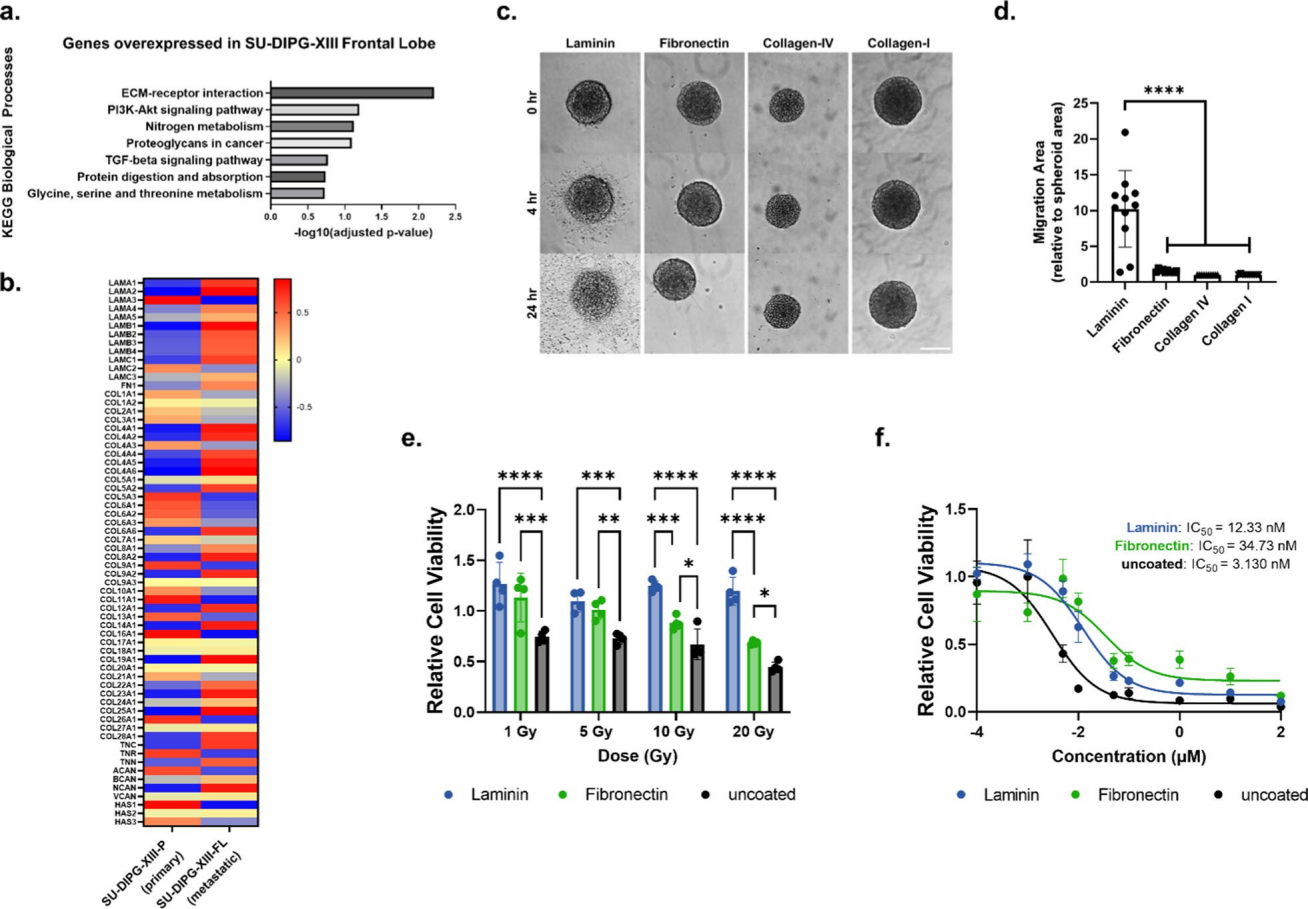
Laminin and, to some extent, fibronectin impact DIPG adhesion, migration, and response to radiation and panobinostat treatment in 2D culture. **a** KEGG enrichment analysis of biological processes highlights ECM-receptor interaction as significantly upregulated in metastatic DIPG in frontal lobe (SU-DIPG-XIII-FL) vs. primary DIPG tumor in the pons (SU-DIPG-XIII-P). **b** Z-score heatmap of average vst-normalized gene expression counts of genes belonging to the ECM family (n = 2/group). **c** Effect of varying ECM coating (laminin, fibronectin, collagen-IV, or collagen-I) on DIPG adhesion and spreading over time. SU-DIPG-XIII-FL spheroids were cultured on 2D tissue culture plastic (TCP) coated with ECM (each at 50 µg/mL), and brightfield time-lapse imaging was performed over 24 h. Scale bar, 200 µm. **d** Quantification of DIPG migration at 24 h. Data is normalized to the spheroid area at 0 h (n ≥ 10 DIPG spheroids per group). ****p < 0.0001 by one-way ANOVA with Dunnett’s multiple comparisons test vs. Laminin. **e, f** Effect of ECM coating on DIPG response to radiation therapy **(e)** or panobinostat **(f)**. Data is reported as relative cell viability (n = 4/group for radiation and n = 3/group for panobinostat treatment). *p < 0.05, **p < 0.01, ***p < 0.001 by two-way ANOVA with Tukey’s multiple comparisons test. Data reported in **d**–**f** represent mean value ± SD

To assess the importance of cell-ECM interactions on DIPG infiltration, we analyzed publicly available RNA sequencing data comparing two DIPG cultures derived from distinct regions of the same patient: (1) SU-DIPG-XIII-P (primary tumor from the pons) and (2) SU-DIPG-XIII-FL (metastatic tumor from the frontal lobe) [3]. Since tumors derived from different patients may have different genomic features, we chose to compare primary vs. metastatic DIPG tumors from the same patient to remove interpatient heterogeneity as a confounding factor. Pathway analysis on significantly upregulated genes (103 genes identified at an FDR < 0.1 with a greater than 4-fold increase) revealed that genes involved in ECM-receptor interactions were enhanced in the metastatic tumor (SU-DIPG-XIII-FL), suggesting that cell-ECM interactions play a role in enabling DIPG infiltration to the frontal lobe from the pons (Fig. 1a). We further found that the gene expression profiles of brain-relevant ECM proteins such as laminin subunits, fibronectin, and collagen-IV subunits were upregulated in the metastatic SU-DIPG-XIII-FL compared to SU-DIPG-XIII-P (Fig. 1b). Immunostaining further corroborated the presence of laminin in both DIPG cell lines, with greater laminin deposition in SU-DIPG-XIII-FL compared to SU-DIPG-XIII-P (Additional file 1: Figure S1).

To identify which ECM proteins support DIPG adhesion and migration, we compared four highly prevalent ECM proteins including laminin, fibronectin, collagen-IV, or collagen-I. SU-DIPG-XIII-FL spheroids were plated on 2D tissue culture plastic (TCP) coated with the respective ECM protein. Within 4 h, laminin enabled robust DIPG cell adhesion and extensive migration out of the spheroids (Fig. 1c, d, Additional file 3: Movie 1, Additional file 4: Movie 2, Additional file 5: Movie 3, Additional file 6: Movie 4). Interestingly, on the fibronectin coating, DIPG spheroids demonstrated a ‘walking’ behavior with the whole spheroid traversing the substrate but minimal cell migration out of the spheroid. This suggests that fibronectin supports DIPG cell adhesion to some extent, but the cell adhesion force to fibronectin is not strong enough to overcome cell–cell adhesion forces within the spheroid. While collagens have been shown to support adult glioma adhesion and spreading, they fail to support DIPG adhesion (Fig. 1c, d, Additional file 3: Movie 1, Additional file 4: Movie 2, Additional file 5: Movie 3, Additional file 6: Movie 4). This highlights that DIPG is distinct from adult brain tumors and needs to be studied independently.

To more thoroughly characterize the variability of ECM gene expression across multiple patient-derived DIPG tumors, we analyzed publicly available RNA sequencing datasets of four additional patient-derived pediatric tumors including three DIPG tumors and one pediatric cortical glioblastoma (pcGBM) (Additional file 1: Figure S2). All additional DIPG cultures (SU-DIPG-VI, SU-DIPG-XXI, SU-DIPG-XXV) demonstrated elevated gene expression of laminin subunits relative to most other ECM proteins (Additional file 1: Figure S2a). We further tested ECM adhesion of three patient-derived DIPG cultures (SU-DIPG-XIII, SU-DIPG-VI, SU-DIPG-XIX) and one pcGBM culture (SU-pcGBM2). SU-DIPG-XIII-P exhibited minimal protrusion on laminin (Additional file 1: Figure S3), indicating upregulated cell-ECM interactions may be uniquely associated with the more invasive DIPG cells (SU-DIPG-XIII-FL). Laminin-mediated adhesion and migration were observed in both SU-DIPG-VI and SU-DIPG-XIX. SU-DIPG-VI also demonstrated modest migration on collagen-I, while SU-DIPG-XIX exhibited modest migration on fibronectin (Additional file 1: Figure S3). Together, these results indicate that laminin is the most robust ECM protein at supporting DIPG adhesion and migration across multiple DIPG cultures.

Interestingly, laminin-mediated adhesion and migration is correlated with an increase in laminin gene expression by qPCR. For the DIPG cell lines that adhered to laminin (SU-DIPG-XIII-FL, SU-DIPG-VI, and SU-DIPG-XIX), we observed significant upregulation of various laminin subunit genes (*LAMA1*, *LAMA2*, *LAMA4*, *LAMB1*, *LAMB2*, and *LAMB3*) compared to SU-DIPG-XIII-P (Additional file 1: Figure S4). However, the specificity of other ECM proteins on supporting DIPG adhesion is heterogeneous and may be patient-specific (Additional file 1: Figure S4). For example, SU-DIPG-VI, with modest migration on collagen-I, exhibited significant upregulation of *COL1A1* compared to SU-DIPG-XIII-P. SU-DIPG-XIII-FL, with modest cell adhesion on fibronectin, exhibited significant upregulation of *FN1* compared to SU-DIPG-XIII-P (Additional file 1: Figures S3 and S4). Further studies are necessary to fully elucidate the role of these cell-ECM interactions.

Next, we investigated how cell-ECM interactions impact DIPG response to standard-of-care radiation therapy in 2D. SU-DIPG-XIII-FL exhibited increased resistance when cultured on laminin- or fibronectin-coated substrates compared to uncoated control (Fig. 1e). At high radiation doses (10 and 20 Gy), laminin coating conferred significantly higher radioresistance compared to fibronectin (Fig. 1e). Using the four additional patient-derived cultures, we further validated that laminin and fibronectin consistently conferred radioresistance to all tested cultures in 2D (Additional file 1: Figure S5a).

We further assessed the impact of ECM adhesion on DIPG response to panobinostat, a promising multiple histone deacetylase (HDAC) inhibitor currently in several clinical trials (NCT02717455, NCT04341311, NCT04804709, and NCT05009992). Compared to uncoated control (IC_50_ = 3.1 nM), both laminin and fibronectin coating enhanced DIPG resistance to panobinostat, with IC_50_ values of 12.3 nM and 34.7 nM, respectively (Fig. 1f). SU-DIPG-XIII-P also exhibited markedly increased drug resistance on fibronectin compared to laminin and uncoated control (Additional file 1: Figure S5b). Relative to uncoated control, SU-DIPG-VI and SU-DIPG-XIX exhibited higher panobinostat resistance on both laminin and fibronectin (Additional file 1: Figure S5b). Together, these data demonstrate that cell-ECM adhesion mediates DIPG migration and resistance to therapies in 2D.

### Laminin-associated integrin knockdown impedes DIPG infiltration in a 3D DIPG-neural assembloid model

**Fig. 2:**
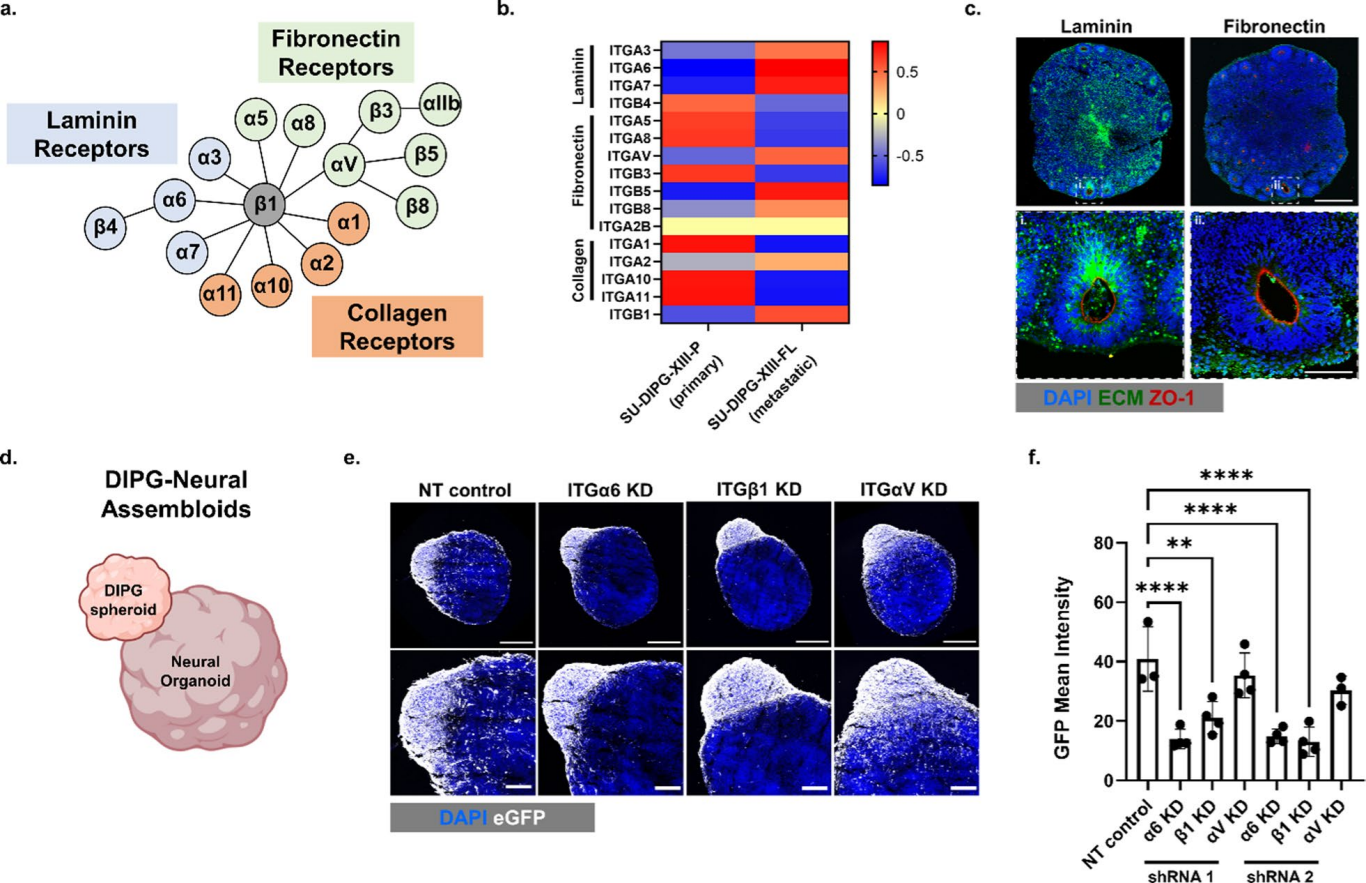
Laminin-associated integrin knockdown impedes DIPG infiltration in a 3D DIPG-neural assembloid model. **a** Schematic of integrin α and β subunit heterodimer combinations known to bind to specific ECM proteins. **b** Z-score heatmap of average vst-normalized gene expression counts of integrin genes (n = 2/group) in SU-DIPG-XIII-P and SU-DIPG-XIII-FL. **c** Representative immunostaining of dorsal forebrain organoids for laminin (green, left panels) or fibronectin (green, right panels), ZO-1 (red), and DAPI (blue). Top panels: scale bar, 500 µm. Bottom panels: scale bar, 100 µm. **d** Schematic of DIPG spheroids fused to dorsal forebrain organoids termed DIPG-neural assembloids. Schematic elements were created using Biorender.com. **e** Representative immunostaining images of DIPG infiltration into DIPG-neural assembloids at day 10 of nontarget (NT) scrambled control DIPG spheroids vs. shRNA integrin knockdown (KD) against ITGα6, ITGβ1, or ITGαV DIPG spheroids. DIPG cells are in pseudocolor (white) for eGFP, DAPI (blue). Top panels: scale bar, 500 µm. Bottom panels: scale bar, 200 µm. **f** Quantification of DIPG infiltration into DIPG-neural assembloids at day 10 based on GFP mean intensity (n ≥ 3 DIPG-neural assembloids per group). **p < 0.01, ****p < 0.0001 by one-way ANOVA with Dunnett’s multiple comparisons test. Data reported in **f** represent mean value ± SD

Cell-ECM adhesions are mediated by transmembrane receptors known as integrins, which form heterodimers between one of 18 α- and one of 8 β-subunits in mammals [9]. The combination of specific subunits enables the binding to different ECM proteins (Fig. 2a). To identify which integrin subunits are involved in the metastatic DIPG cell phenotype, we compared RNA sequencing data between SU-DIPG-XIII-P and SU-DIPG-XIII-FL. The metastatic tumor (SU-DIPG-XIII-FL) showed upregulation of laminin-associated integrins (ITGα3, ITGα6, and ITGα7), fibronectin-associated integrins (ITGαV), and ITGβ1 (ubiquitously involved in multiple interactions) (Fig. 2b**, **Additional file 1: Figure S2c). Similarly, laminin-associated integrins (ITGα6 and ITGα7), along with fibronectin-associated integrins (ITGαV) and ITGβ1, are upregulated across a cohort of patient-derived DIPG cultures including SU-DIPG-XXI, SU-DIPG-XXV, and SU-DIPG-VI (Additional file 1: Figure S2b). This result further highlights the importance of laminin and its associated integrins in DIPG. We note, however, that SU-DIPG-VI expressed a lower level of laminin-associated integrins, indicating that there is some degree of heterogeneity in laminin-associated integrin expression across different patient lines. To probe the role of integrins on DIPG invasion, we generated small hairpin RNA (shRNA)-mediated knockdown against laminin-associated ITGα6, fibronectin-associated ITGαV, or ITGβ1 within the metastatic SU-DIPG-XIII-FL culture. Integrin knockdowns of the SU-DIPG-XIII-FL cell line were validated by flow cytometry (Additional file 1: Figure S6) and qPCR (Additional file 1: Figure S7), which showed consistent trends. Compared to non-target, scrambled shRNA control, knockdown of ITGα6, ITGαV, and ITGβ1 significantly reduced DIPG migration on 2D TCP coated with both laminin and fibronectin, validating the efficacy of disrupting cell adhesions (Additional file 1: Figure S8, Additional file 7: Movie 5, Additional file 8: Movie 6, Additional file 9: Movie 7, Additional file 10: Movie 8).

We next sought to harness a more physiologically relevant 3D model system to validate the role of integrin knockdown on DIPG infiltration. Here, we leverage human induced pluripotent stem cell (hiPSC)-derived neural organoids, which recapitulate key architectural and physiological aspects of brain region-specific features and function [10, 11]. These models provide a unique tool to study human brain development and neurological diseases [10]. Since the metastatic SU-DIPG-XIII-FL culture was collected from the frontal lobe, dorsal forebrain organoids were used in this study. Immunostaining confirmed high expression of laminin throughout the neural organoids, especially within the ventricular zone (VZ)-like structures (indicated by ZO-1 staining) (Fig. 2c), a common site of DIPG infiltration [2, 3]. Fibronectin was also detected, though at a much lower level compared to laminin (Fig. 2c). We recently demonstrated that DIPG spheroids can be fused to neural organoids to form DIPG-neural assembloids to mimic DIPG infiltration in the brain. DIPG exhibited rapid infiltration into the neural organoid within 7 days, demonstrating its utility as a physiologically relevant model to study DIPG infiltration [12]. Furthermore, we observed that the metastatic DIPG cell line (SU-DIPG-XIII-FL) demonstrated significantly enhanced infiltration within the DIPG-neural assembloid model compared to the primary tumor (SU-DIPG-XIII-P) (Additional file 1: Figure S9). This result further supports the physiological relevance of the DIPG-neural assembloid model in retaining the patient tumor biology.

To assess the role of integrin knockdown on DIPG infiltration, we fused shRNA nontarget control and shRNA integrin knockdown DIPG spheroids to neural organoids (Fig. 2d). Remarkably, ITGα6 and ITGβ1 knockdown significantly reduced DIPG infiltration into the neural organoids 10 days after fusion (Fig. 2e, f, Additional file 1: Figure S10). While ITGαV knockdown reduced migration on 2D surfaces (Fig. 2c), no significant reduction in DIPG infiltration was observed within the 3D DIPG-neural assembloid (Fig. 2e, f), suggesting the importance of using 3D models to study the role of integrin receptors on DIPG infiltration. Together, these results demonstrate that disrupting laminin-associated integrins (ITGα6 and ITGβ1) can significantly halt DIPG infiltration in a physiologically relevant DIPG-neural assembloid model.

### Laminin-associated integrin knockdown improves DIPG response to radiation therapy

**Fig. 3:**
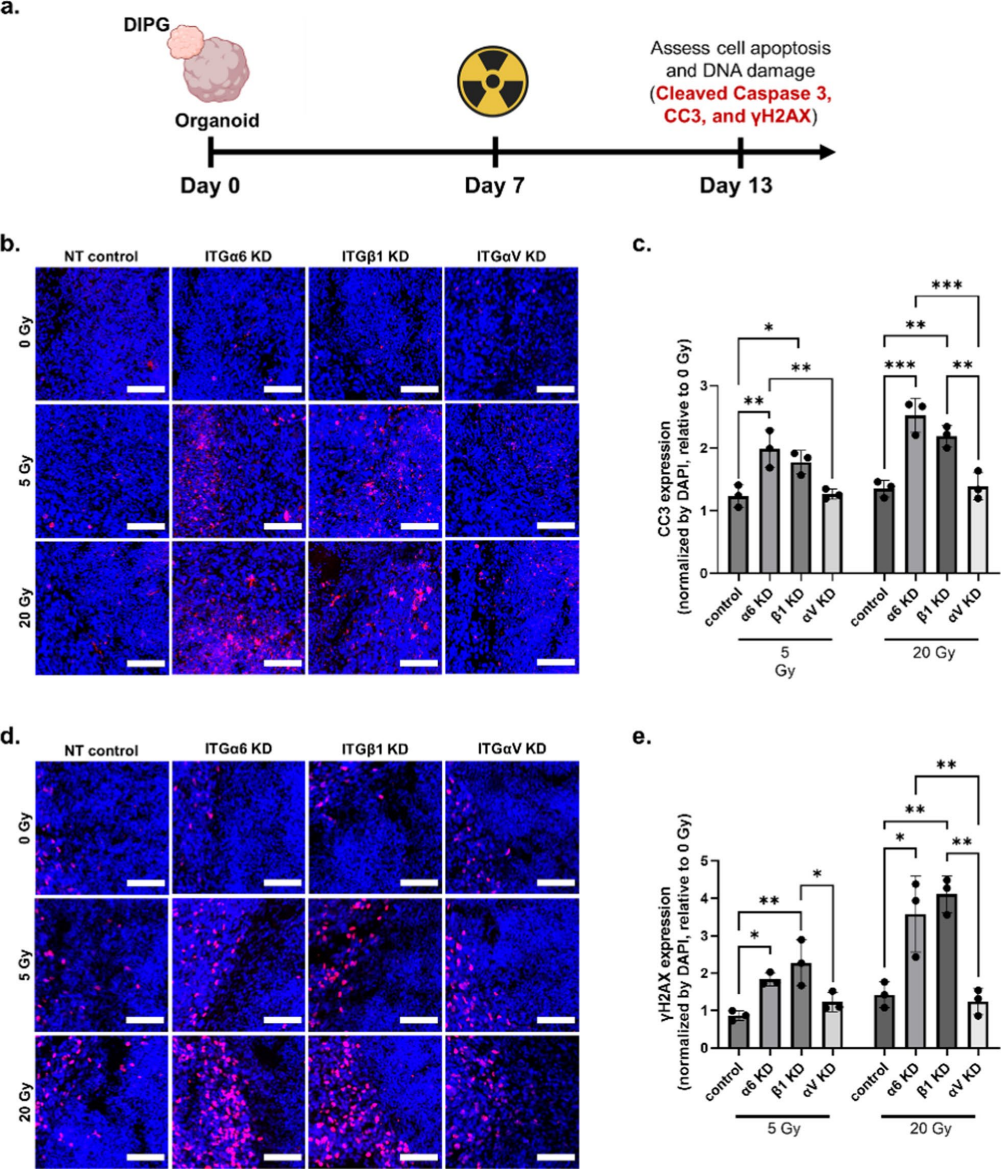
Laminin-associated integrin knockdown improves DIPG response to radiation in a 3D DIPG-neural assembloid model. **a** Schematic of experimental design to assess the role of integrin knockdown (KD) on radiation response within DIPG-neural assembloids. **b** Representative immunostaining for Cleaved Caspase 3 (CC3, red) and DAPI (blue) at the fusion interface within the DIPG-neural assembloids when irradiated at 0, 5, and 20 Gy. Scale bar, 100 µm. **c** Quantification of the relative degree of apoptosis based on CC3 immunostaining at the fusion interface normalized by DAPI and relative to the untreated assembloid control group in each respective KD group (n = 3 assembloids per group). **d** Representative immunostaining for γH2AX (red) and DAPI (blue) at the fusion interface within the DIPG-neural assembloids when irradiated at 0, 5, and 20 Gy. Scale bar, 100 µm. **e** Quantification of the relative degree of DNA damage based on γH2AX immunostaining at the fusion interface normalized by DAPI and relative to the untreated assembloid control group in each respective KD group (n = 3 assembloids per group). *p < 0.05, **p < 0.01, ***p < 0.001 by one-way ANOVA with Tukey’s multiple comparisons test. Data reported in **c** and **e** represent mean value ± SD

Radiation therapy is the only standard-of-care for DIPG patients and can merely extend patient survival by about three months [1]. Driven by the desperate need to improve treatment outcomes for DIPG patients, we next sought to investigate whether integrin knockdown can help improve DIPG response to radiation therapy. Given that DIPG-laminin interactions significantly enhanced radioresistance in 2D (Fig. 1e), we hypothesized that the knockdown of laminin-associated integrins would increase DIPG susceptibility to radiation therapy. On 2D TCP coated with both laminin and fibronectin, integrin knockdown of ITGα6, ITGβ1, and ITGαV improved DIPG radiation response at all tested dosages (Additional file 1: Figure S11). To further validate the effect of laminin-associated integrin knockdown on DIPG radiation response in 3D, DIPG-neural assembloids were irradiated 7 days after fusion and fixed on day 13 to assess cell apoptosis and DNA damage by Cleaved Caspase 3 (CC3) and γH2AX expression, respectively (Fig. 3a). Minimal cell apoptosis and DNA damage were observed within nontarget control treated assembloids at even the highest dosage (20 Gy) of radiation (Fig. 3b–e), indicating high resistance to radiation therapy. In contrast, knockdown of ITGα6 and ITGβ1 resulted in significantly higher levels of CC3 and γH2AX expression compared to nontarget control (Fig. 3b–e). Radiation generally works more effectively in fast-dividing cells and should decrease cell proliferation post-radiation [13]. To evaluate if the differences in radiation sensitivity are associated with changes in cell proliferation, we characterized cell proliferation post-radiation using Ki67 staining. At both doses post-radiation, integrin knockdown of ITGα6 and ITGβ1 significantly reduced cell proliferation compared to the control (Additional file 1: Figure S12). This result confirms that the integrin knockdown-induced radiation sensitivity is accompanied by decreased cell proliferation. ITGαV knockdown improved radiation response in 2D (Additional file 1: Figure S5c), but no improvement was observed within the DIPG-neural assembloid even at 20 Gy (Fig. 3b–e). Together, these results provide strong evidence that blocking DIPG-laminin interactions can help overcome radioresistance and improve DIPG response to radiation therapy.

### ITGα6 knockdown improves DIPG response to panobinostat

**Fig. 4:**
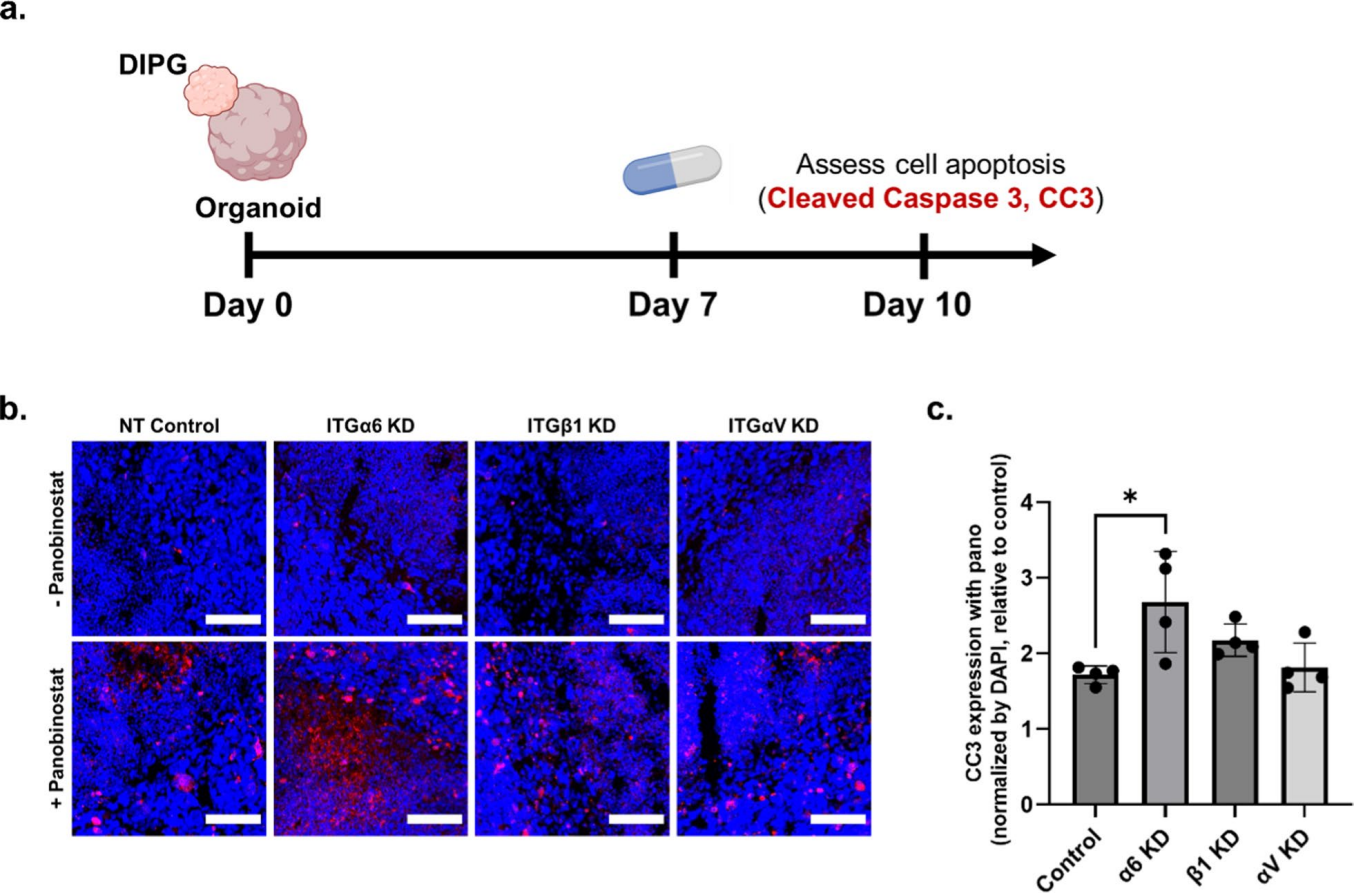
Integrin α6 knockdown improves DIPG drug response to panobinostat. **a** Schematic of experimental design to assess the role of integrin knockdown (KD) on panobinostat response within DIPG-neural assembloids. **b** Representative immunostaining for Cleaved Caspase 3 (CC3, red) and DAPI (blue) at the fusion interface within the DIPG-neural assembloids when treated with 0 or 200 nM panobinostat. Scale bar, 100 µm. **c** Quantification of the relative degree of apoptosis based on CC3 immunostaining at the fusion interface normalized by DAPI and relative to the untreated assembloid control group in each respective KD group (n = 3 assembloids per group). *p < 0.05 by one-way ANOVA with Tukey’s multiple comparisons test. Data reported in **c** represent mean value ± SD

While panobinostat has been identified as a promising therapeutic, DIPG cells ultimately develop resistance [8]. We hypothesized that combinatorial targeting of integrin knockdown and HDAC inhibition would lead to higher DIPG cell death by disrupting two distinct oncogenic pathways. Integrin knockdown of ITGα6, ITGβ1, and ITGαV offered a modest improvement in panobinostat response in 2D culture coated with laminin and fibronectin (Additional file 1: Figure S13). To more robustly assess this, we treated DIPG-neural assembloids with panobinostat 7 days after fusion and fixed them 3 days later to assess cell apoptosis by CC3 expression (Fig. 4a). All groups exhibited increased cell apoptosis by CC3 expression relative to the respective untreated control. Compared to nontarget control, only knockdown of ITGα6 resulted in significantly higher levels of CC3 expression (Fig. 4b, c). These data indicate that ITGα6 is a promising target for combinatorial therapy to further increase DIPG response to panobinostat. Interestingly, while ITGβ1 knockdown improved radiation resistance (Fig. 3), no benefit was seen in improving response to panobinostat (Fig. 4), suggesting that different therapies may be uniquely regulated by distinct cell-adhesive interactions and specific integrin subunits.

## Discussion

By harnessing a DIPG-neural assembloid model, we demonstrate that disrupting cell-ECM adhesions can be a novel therapeutic strategy to extend patient survival. Specifically, targeting laminin-associated integrins reduced DIPG infiltration and enhanced the efficacy of current treatment options within DIPG-neural assembloids. Among the four ECM proteins we tested, laminin, which is present throughout the brain [14], supports robust DIPG adhesion and migration across multiple DIPG cultures. DIPG cells are reminiscent of neural precursor cells (NPCs) [15] and likely originate from oligodendrocyte precursor cells [4], which have been found to be tightly regulated by laminin [16]. Moreover, laminin plays a critical role in regulating the maintenance of neural stem cells and glioblastoma stem cells [17, 18]. Interestingly, DIPG cells preferentially invade into the stem cell-rich subventricular zone [2, 3], in which laminin provides adhesive and regulatory cues for the stem cell populations [19]. Previous work showed that interactions with neural progenitor cells drive DIPG spread to the subventricular zone [3]. Our work suggests ECM adhesion is another mechanism that facilitates DIPG infiltration to the subventricular zone. Neural organoids recapitulate VZ-like structures [11], and we observed high laminin expression in these regions in our neural organoid model (Fig. 2e). There are a number of ECM components and integrins that are not upregulated in the metastatic cell line. We also note that certain collagen-related integrins are upregulated in the primary DIPG line (SU-DIPG-XIII-P) compared to the metastatic DIPG line (SU-DIPG-XIII-FL). Future studies could explore the roles of these additional ECM proteins in DIPG progression by knocking down or overexpressing these components within this DIPG-neural assembloid model to further study various mechanisms that drive DIPG infiltration to subventricular zones.

Previous work has shown that ITGα6 promotes radioresistance in adult GBM by modulating DNA damage response [20]. DIPG is a distinct pediatric glioma, and our results provide the first evidence that targeting laminin-associated integrins also impacts DIPG radioresistance. Moreover, ITGα6 knockdown enhances DIPG response to panobinostat in DIPG-neural assembloid models, highlighting the synergy of targeting two distinct oncogenic pathways (one at the cell-ECM level and one at the epigenetic level) to improve DIPG treatment outcomes. Emerging work has revealed that integrin-mediated cell–matrix interactions can lead to epigenetic changes [21]. Future work can investigate how integrin knockdown may mechanistically synergize with HDAC inhibition.

Another key finding of this study is the importance of using 3D DIPG-neural assembloid models to validate the efficacy of new therapeutic strategies that target cell-ECM adhesions. For example, while ITGαV knockdown improved radiation and panobinostat response in 2D, no benefit was seen within the 3D DIPG-neural assembloid. It is well established that 2D culture often fails to retain tumor biology of patient tumors or preclinical models, whereas 3D models are more faithful in predicting drug responses [22, 23]. Our results establish targeting specific integrins as a promising strategy to improve tumor responses to standard therapies such as radiation in 3D models. Like any in vitro model, we recognize the assembloid model cannot recapitulate all the complexity of patient tumors in vivo, and it is possible the importance of ECM components in therapeutic response could also differ between the 3D assembloid model and in vivo. Future studies may further validate the effect of targeting specific integrins in modulating DIPG response to radiation therapy using in vivo animal models.

Furthermore, our results provide evidence that targeting ITGα6 is effective in reducing DIPG infiltration and enhancing responses to radiation and panobinostat. To date, most available integrin inhibitors target ITGαV, but not laminin-associated integrins [24]. Our results motivate future studies on developing small molecule inhibitors against ITGα6 as a potential therapy for DIPG. To minimize off-target effects to other cell types, consideration must be given to achieve specific inhibition of DIPG cells in vivo [18, 19]. Lastly, we utilized a dorsal forebrain organoid in this study. DIPG is known to widely disseminate throughout the brain. Advancements in neural organoid protocols now enable the formation of various regionalized brain organoids mimicking the midbrain, ventral forebrain, thalamus, and spinal cord [11, 25–27]. Therefore, future studies may fuse DIPG spheroids to other regionalized neural organoids to investigate how ECM interactions within a particular brain region mediate DIPG infiltration and therapy responses.

## Methods

### Primary DIPG and pcGBM culture

Patient-derived primary DIPG and pcGBM cultures (SU-DIPG-XIII-FL, SU-DIPG-XIII-P, SU-DIPG-VI, SU-DIPG-XIX, and SU-pcGBM2), obtained at the time of biopsy or autopsy, were provided by Dr. Michelle Monje at Stanford University. All human tumor cell cultures were generated with informed consent and under institutional review board (IRB)-approved protocols, as previously reported [3, 4, 8]. All patient-derived cultures were grown as tumor neurospheres in tumor stem medium consisting of DMEM/F12 (Invitrogen, 11330032), Neurobasal(-A) (Thermo Fisher Scientific, 10888022), B-27 supplement without vitamin A (1:50, Thermo Fisher Scientific 12587010), human EGF (20 ng ml^−1^, Shenandoah Biotech 100–26), human b-FGF (20 ng ml^−1^, Shenandoah Biotech 100–146), human PDGF-AA (10 ng ml^−1^, Shenandoah Biotech 100–16), human PDGF-BB (10 ng ml^−1^, Shenandoah Biotech 100–18), and heparin (2 µg ml^−1^, StemCell Tech 07980). Media was changed once per week. See Additional file 1: Table S1 for details of the patient-derived cultures used in this study.

### hiPSC culture

All human induced pluripotent stem cells (hiPSCs) were previously validated with respect to their stemness and differentiation capacity [28]. All hiPSCs were tested for and maintained mycoplasma free. Approval for this study was obtained from the Stanford IRB, and informed consent was obtained from all donors. hiPSCs were maintained in their pluripotent state by being cultured with mTeSR Plus media (StemCell Tech 100-0276) in monolayer on hESC-qualified Matrigel (0.1 mg ml^−1^, Sigma 354277).

### Neural organoid differentiation and maturation

Dorsal forebrain organoids were differentiated according to previously published protocols [11, 29]. Briefly, hiPSCs were dissociated with Accutase (StemCell Tech 07920) and aggregated into uniform 5000 cell aggregates with AggreWell800 plates (StemCell Tech 34815) in mTeSR Plus media supplemented with ROCK inhibitor Y-27632 (10 µM, Selleckchem S1049). After 24 h, hiPSC aggregates were transferred to ultra-low attachment culture dishes (Corning 4615) in Essential 6 medium (Thermo Fisher A1516401) supplemented with the two dual SMAD inhibitors SB-431542 (10 µM, Tocris 1614) and LDN-193189 (100 nM, StemCell Tech 72147). Media was changed daily. On day 6 of differentiation, neural organoids were transferred to neural medium consisting of Neurobasal(-A) (Thermo Fisher 10,888,022), B-27 Supplement without vitamin A (1:50, Thermo Fisher 12587010), GlutaMAX Supplement (1:100, Thermo Fisher 35050079), Penicillin–Streptomycin (1:100, Thermo Fisher 15070063), and supplemented with human EGF (20 ng ml^–1^, PeproTech AF-100–15) and human FGF-2 (20 ng ml^–1^, PeproTech AF-100-18B). From day 25 to 42, neural medium was supplemented with the growth factors BDNF (20 ng ml^–1^, PeproTech AF-450–02) and NT3 (20 ng ml^–1^, PeproTech AF-450-03), and media was changed every other day. From day 43 onward, neural organoids were maintained in neural medium with media changes every four days.

### Generation of DIPG-neural assembloids

To generate DIPG-neural assembloids, dorsal forebrain organoids (between days 43–80) and DIPG spheroids were generated separately and then assembled by placing them in close proximity at the bottom of a 1.5 mL Eppendorf tube in neural medium in an incubator. DIPG-neural assembloids fused within 24 h and were transferred to 24-well plates using a cut p1000 pipette tip. Media was changed every 4 days.

### Assessing DIPG adhesion and migration in 2D

Tissue culture plastic (TCP) was coated with laminin (50 µg mL^−1^, Sigma-Aldrich L2020), fibronectin (50 µg mL^−1^, Fisher Scientific CB-40008A), collagen-I (50 µg mL^−1^, Corning 354236), or collagen-IV (50 µg mL^−1^, Sigma-Aldrich C0543) solutions for 2 h at 37 °C. Solutions for laminin and fibronectin were dissolved in DPBS; solutions for collagen-I and collagen-IV were dissolved in 0.1% acetic acid. Wells were subsequently washed 3× with DPBS. DIPG spheroids were then placed on coated TCP to assess adhesion and migration. Images were collected with a Keyence BZ-800 with a 10X objective shortly after seeding the DIPG spheroids, 0 h, and at 24 h. To quantify migration, ImageJ was used to measure the migration area at 24 h, which was normalized to the spheroid area at 0 h. For live-cell time-lapse imaging to monitor DIPG adhesion and migration, a DMi8 inverted epifluorescence microscope (Leica) with a 20X objective and equipped with a live-cell incubator was used. Images were captured every 10 or 20 min for 24 h.

### Generation of integrin knockdown cell lines and characterization using flow cytometry

shRNA constructs against ITGα, ITGβ1, and ITGαV (Sigma Aldrich) and nontarget scrambled shRNA construct (generously gifted by the laboratory of Dr. Michelle Monje) were packaged into lentiviral particles by the Neuroscience Viral Vector Core at Stanford University. For lentivirus infection, dissociated SU-DIPG-XIII-FL cells were exposed to shRNA-expressing lentivirus for 12–16 h before replacing with fresh medium to allow cells to recover. After 48 h, puromycin (Sigma Aldrich P8833) was added to select positively infected DIPG cells. After puromycin selection, DIPG cells were grown for at least one passage before using in experiments. The shRNA constructs are provided in Additional file 1: Table S2.

To assess knockdown efficacy, SU-DIPG-XIII-FL cells transfected with shRNA-expressing lentivirus were characterized by flow cytometry. Cells were dissociated, permeabilized, and stained for intracellular and extracellular protein expression. The following antibodies were used to assess ITGα6, ITGβ1, and ITGαV expression: ITGα6 (PE-CF594 Rat Anti-Human, BD bioscience 562493), ITGαV (PE anti-human CD51, BioLegend 327909), and ITGβ1 (BV786 Mouse Anti-Human CD29, BD bioscience 564815) expression. Results were analyzed by FlowJo Software.

### Radiation and panobinostat treatment on 2D and within DIPG-neural assembloids

For 2D studies, DIPG and pcGBM neurospheres were dissociated, and single cells were seeded on ECM coated 96 well plates at 1000 cells/well and allowed to adhere overnight. Plates were coated with either laminin (50 µg mL^−1^, Sigma-Aldrich L2020), fibronectin (50 µg mL^−1^, Fisher Scientific CB-40008A), or laminin and fibronectin (each at 50 µg mL^−1^). Cell treatment started one day after seeding. For radiation studies, cells were irradiated using a SmART cabinet X-ray irradiator (Precision X-Ray). A single beam was targeted at the cells, delivering the following doses: 1, 5, 10, and 20 Gy. Cells were cultured for an additional 5 days, at which point Presto Blue assay (Thermo Fisher Scientific P50201) was used to measure the cell viability. For panobinostat (Selleckchem S1030) treatment, cells were treated at a concentration range from 0.1 nM to 100 µM. Cell viability was measured at day 3 after panobinostat treatment. Relative cell viability was calculated by normalizing treatment groups to the untreated control.

For radiation treatment of the DIPG-neural assembloids, the assembloids was irradiated at 5 Gy and 20 Gy as described above on day 7. Assembloids were cultured for 6 more days, after which samples were fixed in 4% paraformaldehyde (PFA, Electron Microscopy Sciences 15700) in DPBS for immunohistochemistry. For panobinostat treatment, DIPG-neural assembloids were cultured first for 7 days, then treated with 200 nM panobinostat for 3 more days before harvest. Samples were fixed in 4% PFA in DPBS for immunohistochemistry.

### Immunohistochemistry

Organoids and assembloids were fixed in 4% PFA for 2 h at 4 °C. They were then washed in DPBS three times for 15 min each and transferred to a 30% sucrose solution in DPBS for 2–3 days at 4 °C. Once the organoids or assembloids sank in the sucrose solution, they were embedded in a 1:1 mixture of OCT (Fisher Scientific 23-730-571) and 30% sucrose in DPBS and snap-frozen using dry ice. For immunostaining, 40 µm sections were cut using a Leica cryostat. Cryosections were washed with DPBS to remove excess OCT and permeabilized with 0.25% Triton X-100 (Thermo Fisher A16046) in DPBS (DPBS-T) for 1 h at room temperature (RT). They were then blocked in 5% goat serum (Gibco 16210-072), 5% bovine serum albumin (BSA, Sigma A9418), and 0.5% Triton X-100 in DPBS for 3 h at RT. The sections were then incubated overnight at 4 °C with primary antibodies diluted in 2.5% goat serum, 2.5% BSA, and 0.5% Triton X-100 in DPBS. To facilitate visualizing infiltrating DIPG cells into the assembloid, DIPG cells were labelled with GFP and also stained with H3K27M, a marker for DIPG. The following primary antibodies were used including GFP (rabbit, 1:200, Thermo Fisher A11122), cleaved caspase-3 (rabbit, 1:400, Cell Signaling 9661), gamma H2A.X (mouse, 1:200, Abcam ab26350), laminin (rabbit, 1:300, Abcam ab11575), fibronectin (rabbit, 1:100, Invitrogen PA1-23693), ZO-1 (mouse, 1:150, Invitrogen 33-9100), histone H3 (mutated K27M) (rabbit, 1:400, Abcam ab190631), Ki67 (rabbit, 1:200, Abcam ab16667). DPBS-T was used to wash the samples three times for 30 min each, and the samples were then incubated overnight at 4 °C with secondary antibodies Alexa Fluor 488 (1:500, Thermo Fisher A-11034) and Alexa Fluor 647 (1:500, Thermo Fisher A-21236) and 4′,6-diamidino-2-phenylindole (DAPI, 5 mg mL^−1^ stock, 1:2000, Thermo Fisher 62247) in the same antibody dilution solution. The next day, samples were washed with DPBS-T three times for 20 min each and mounted to No. 1 glass coverslips with ProLong Gold Antifade Reagent (Cell Signaling 9071). Samples were imaged using a Leica STELLARIS 5 confocal microscope with a 10X or 63X objective.

### RNA-sequencing analysis

Transcriptome data (RNA-seq) of DIPG cell cultures derived from SU-DIPG-XIII-P and SU-DIPG-XIII-FL were downloaded from the GEO dataset with accession ID: GSE99812. SU-DIPG-XIII-P and SU-DIPG-XIII-FL cell cultures used to generate the RNA sequencing data sets were cultured under similar conditions as reported above. Trimmed reads were aligned to the Human reference genome hg38 using the RNA STAR aligner. featureCounts was used to determine read abundance and generate count files. Differentially expressed genes (DEGs) were determined using DESeq2 [27]. DEGs with an adjusted *p*-value of less than 0.1 were considered significantly different and used for subsequent pathway analysis using the EnrichR tool [28]. Heatmaps depicting gene expression profiles were made using the average z-scores of vst-normalized RNA-seq counts obtained from DESeq2. Count files for the SU-DIPG-VI, SU-DIPG-XXI, SU-DIPG-XXV and SU-pcGBM2 cell lines were downloaded from GEO datasets with accession IDs GSE222481, GSE222560, and GSE99045. TPM (transcripts per million) were extracted and log values were used to generate plots comparing transcript abundance across all cell lines. See Additional file 2: Table S4 for mean gene expression values, log2(fold-change), *p*-values, and adjusted *p*-values (False Discovery Rate).

#### Quantitative polymerase chain reaction (qPCR)

DIPG and pcGBM spheroids were dissociated, immediately resuspended in TRIzol reagent (Invitrogen 15596018), and frozen at −80 °C until use. mRNA was purified from lysates using a phenol–chloroform extraction. Samples were first disrupted by probe sonication (50% amplitude (25 watts), 30 kHz frequency, 0.5 s cycle), transferred to phase lock gels (Quantabio 5PRIME 2302830), and 100 µL of chloroform (Sigma CX1055) was added to each sample. Samples were then centrifuged at 15,300×*g* for 15 min at 4 °C, and the aqueous phase was transferred to a clean 1.5-mL microcentrifuge tube. Samples were precipitated with isopropyl alcohol and washed twice with 70% ethanol, with centrifugation steps between each wash (18,500×*g* for 10 min at 4 °C). Samples were then dried and resuspended in 15 µL of nuclease-free water. mRNA concentrations were measured on a NanoDrop (Thermo Scientific), and 800 ng mRNA of each sample was reverse transcribed using a High-Capacity cDNA Reverse Transcription Kit (Applied Biosystems 4368814). For each gene target, qPCR was performed on 6.6 µL of diluted cDNA mixed with 0.9 µL of 5 µM forward and reverse primer pair solution and 7.5 µL of Fast SYBR Green Master Mix (Applied Biosystems 4385612). Samples were run on a StepOnePlus Real Time PCR System (Applied Biosystems). Cycle threshold (CT) values were calculated using the StepOnePlus software (v.2.3) and normalized to GAPDH as a housekeeping gene (∆CT). Statistical analysis was performed before transforming to a natural scale, and relative mRNA expression is reported as a geometric mean with standard deviation. See Additional file 1: Table S3 for information about qPCR primers. Melt curves were performed for all primer pairs.

#### Image analysis

*Quantification of CC3 and γH2AX expression:* Images obtained from confocal microscopy were processed using a custom Python script to extract the pixel-by-pixel total sum of the red and blue channels that corresponded to the DAPI and CC3 or γH2AX signals, respectively. The reported CC3 or γH2AX expression was calculated by dividing the total CC3 or γH2AX signal by the total DAPI signal and normalizing it to the untreated group in each shRNA integrin knockdown cell line.

*Quantification of DIPG infiltration:* A custom Python script was used to assess the extent of DIPG infiltration from images obtained from confocal microscopy. Briefly, after performing a maximum intensity z-projection to produce a 2D image, a graphical interface allowed the user to draw a closed boundary around the neural organoid, separating the organoid from the DIPG spheroid. The boundaries were saved for repeated use, and the drawer ensured the use of consistent criteria for delineating the organoid edge. Afterwards, a point-in-polygon test was leveraged to determine which points lie inside the boundary and the minimum Euclidian distance to the boundary for each interior point. Hence, the algorithm assigned each point on the interior a minimum distance from the boundary and subsequently extracted its corresponding intensity for each signal channel. These distances were used to bin the points into equally-spaced concentric shells that signify escalating “levels” of infiltration towards the organoid center. Additionally, a metric for total infiltration either past a certain micrometer distance from the boundary or a percentage distance towards the organoid center were computed. After determining the points that fall within each level or past a certain threshold, the mean intensity of each marker was computed from all points in that group, and the reported infiltration metrics were calculated by normalizing the average eGFP signal to the average DAPI signal. To quantify infiltration by H3K27M staining, a maximum intensity z-projection was performed to produce a 2D image, after which a straight line was drawn orthogonal from the neural organoid boundary to the H3K27M + cell to determine the infiltration distance.

#### Statistical analysis

Statistical analyses were performed using GraphPad Prism Software. Specifics on statistical tests used and the corresponding p and n values are provided with each of the figure legends.

### Supplementary Information

**Additional file 1**. Supplementary information.

**Additional file 2**. RNA sequencing analyses details and parameters.

**Additional file 3**. **Supplementary Movie 1:** SU-DIPG-XIII-FL spheroid on laminin-coated TCP.

**Additional file 4**. **Supplementary Movie 2:** SU-DIPG-XIII-FL spheroid on fibronectin-coated TCP.

**Additional file 5**. **Supplementary Movie 3:** SU-DIPG-XIII-FL spheroid on collagen-IV-coated TCP.

**Additional file 6**. **Supplementary Movie 4:** SU-DIPG-XIII-FL spheroid on collagen-I-coated TCP.

**Additional file 7**. **Supplementary Movie 5:** Non-target, scrambled shRNA control SU-DIPG-XIII-FL spheroid on laminin- and fibronectin-coated TCP.

**Additional file 8**. **Supplementary Movie 6:** shRNA mediated ITGα6 knockdown of SU-DIPG-XIII-FL spheroid on laminin- and fibronectin-coated TCP.

**Additional file 9**. **Supplementary Movie 7:** shRNA mediated ITGβ1 knockdown of SU-DIPG-XIII-FL spheroid on laminin- and fibronectin-coated TCP.

**Additional file 10**. **Supplementary Movie 8:** shRNA mediated ITGαV knockdown of SU-DIPG-XIII-FL spheroid on laminin- and fibronectin-coated TCP.

## Data Availability

The data that support the findings of this study are available from the corresponding author upon reasonable request.
