# Supplementary material for: Laminin-associated integrins mediate Diffuse Intrinsic Pontine Glioma infiltration and therapy response within a neural assembloid model
Source: Acta Neuropathol Commun. 2024 May 5;12:71. doi: 10.1186/s40478-024-01765-4 (PMC11070088; doi:10.1186/s40478-024-01765-4)
Supplement: Supplementary file 1 — Additional file 1. Supplementary information. [file 40478_2024_1765_MOESM1_ESM.docx]

**Supplementary information**

**Laminin-associated integrins mediate Diffuse Intrinsic Pontine Glioma infiltration and therapy response within a neural assembloid model**

Sauradeep Sinha,^1†^ Michelle Huang,^2†^ Georgios Mikos,^2^ Yudhishtar Bedi,^3^ Luis Soto,^4^ Sarah Lensch,^1^ Manish Ayushman,^1^ Lacramioara Bintu,^1^ Nidhi Bhutani,^3^ Sarah Heilshorn,^5*^ Fan Yang^1,3*^


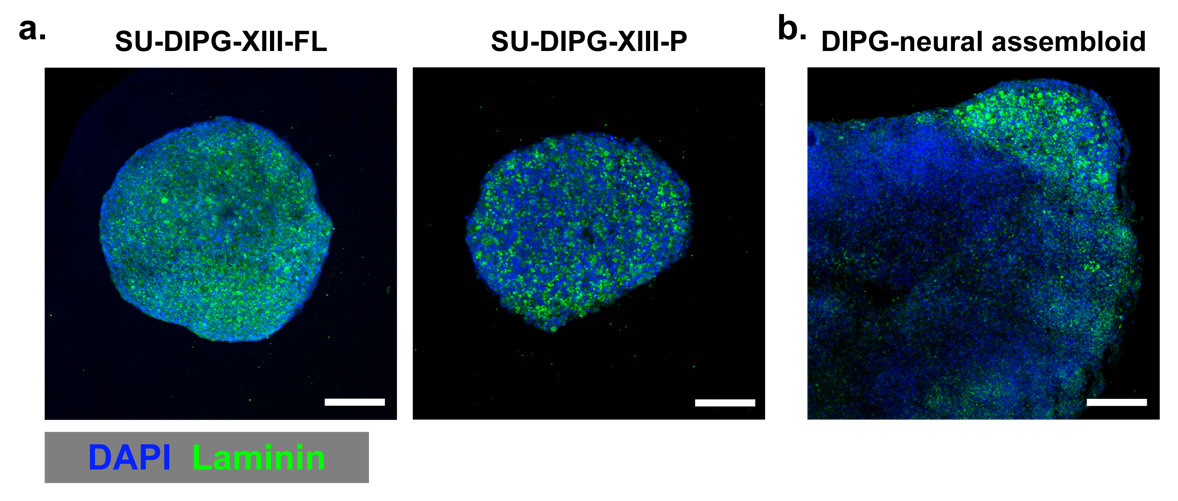


**Supplementary Fig. 1. Laminin deposition within DIPG cultures.** Representative immunostaining of patient-derived DIPG spheroids **(a)** and a DIPG-neural assembloid composed of SU-DIPG-XIII-FL fused to a neural organoid **(b)** for laminin (green) and DAPI (blue). Scale bar, 200 µm.

**
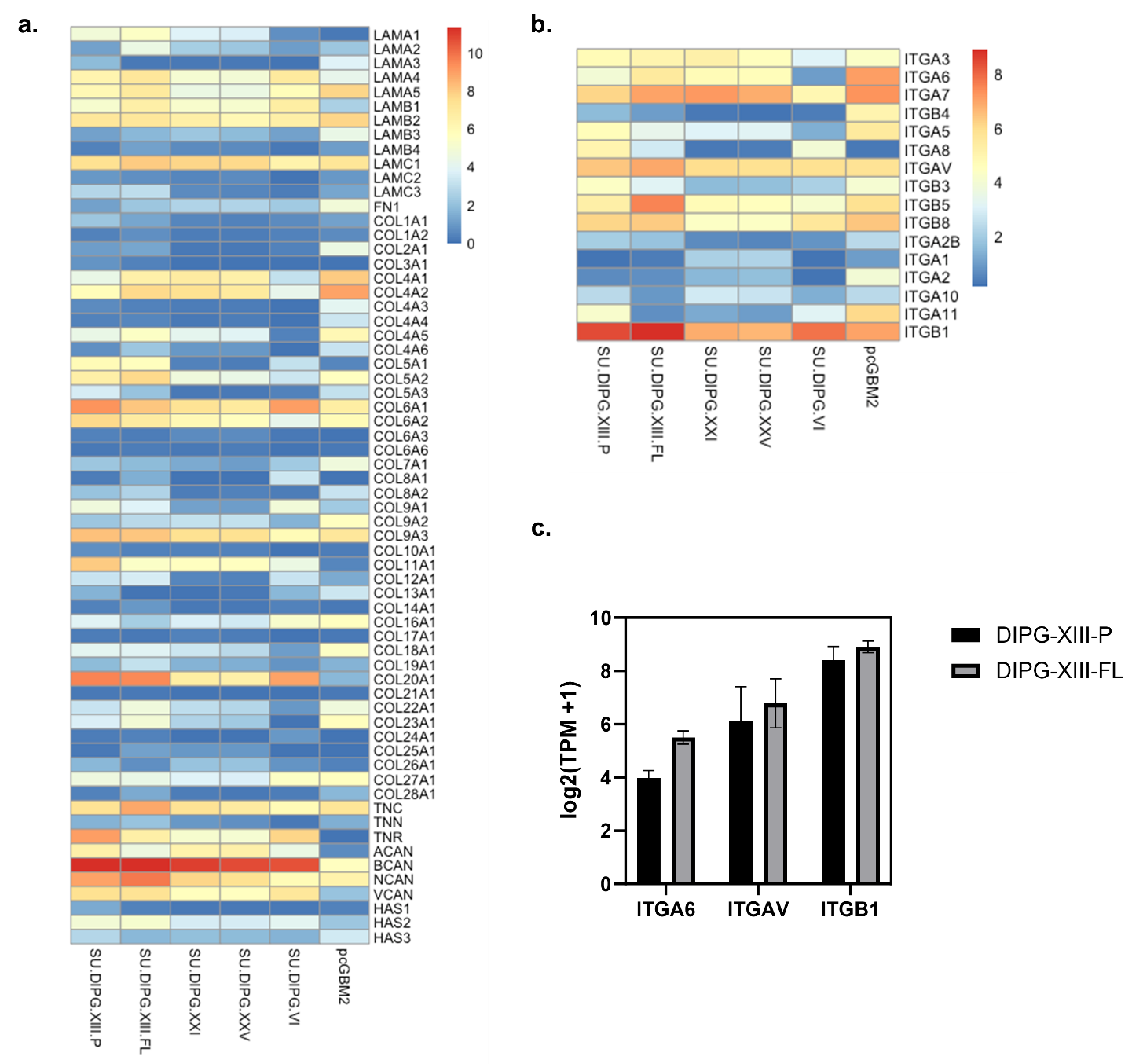
**

**Supplementary Figure 2.** **RNA sequencing analyses of ECM components and integrin subunits across six patient-derived pediatric tumor cultures.** Heatmaps depicting the transcript abundance of ECM gene markers **(a)** and integrin genes **(b)** across all glioma cell lines. **(c)** Bar plot depicting relative transcript abundance of integrins knocked down in this study (*ITGA6*, *ITGAV* and *ITGB1*) in the SU-DIPG-XIII-P and SU-DIPG-XIII-FL lines. All plots depict the log2(TPM+1) values of the genes displayed.


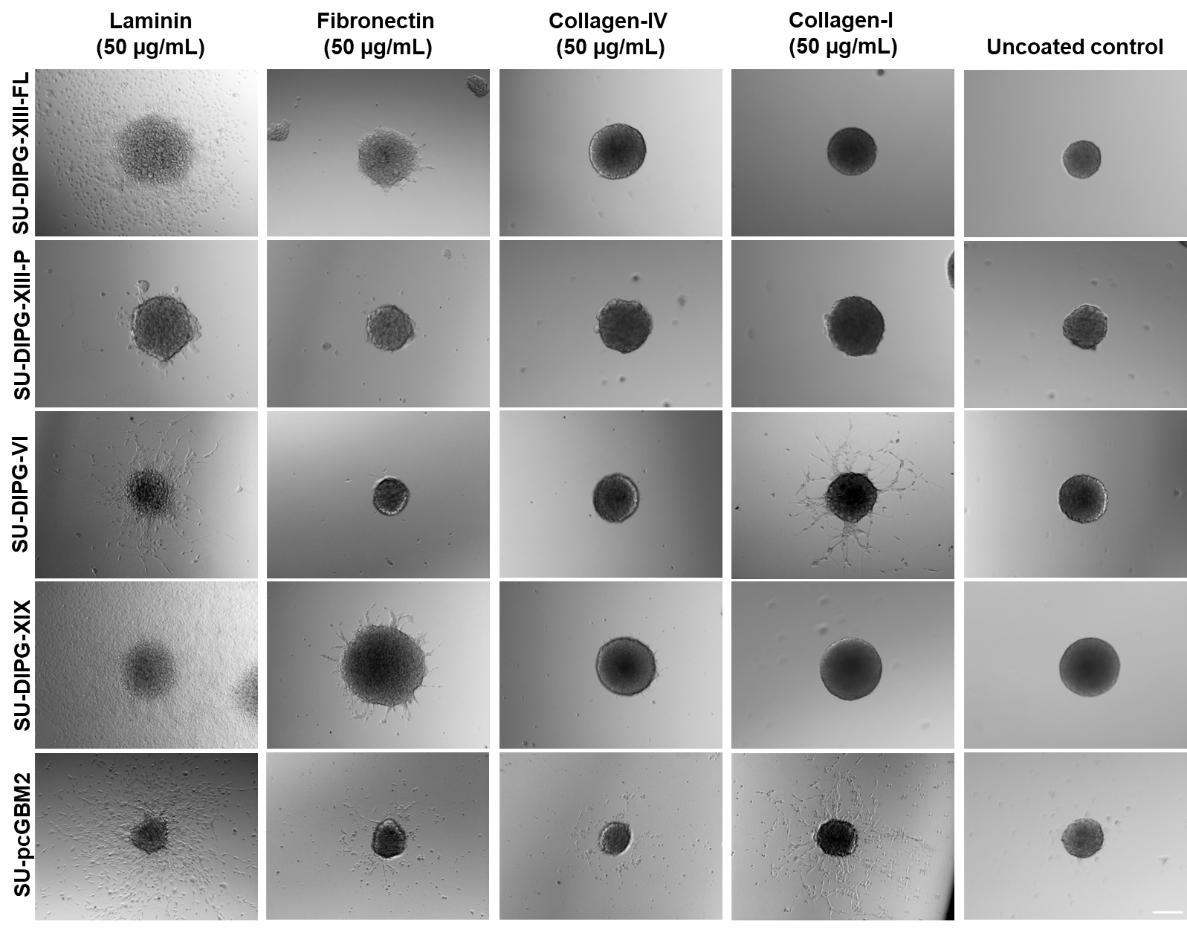


**Supplementary Figure 3. Assessing the effect of varying ECM coating on the adhesion and spreading of four patient-derived DIPG cell lines and one pcGBM cell line in 2D.** Brightfield imaging of patient-derived DIPG or pcGBM spheroids cultured on 2D tissue culture plastic (TCP) coated with laminin, fibronectin, collagen-IV, or collagen-I. ECM coating concentration was kept constant at 50 µg/mL. Uncoated TCP was included as a control. All data was collected at 24 hrs after cell plating. Scale bar, 100 µm.

**
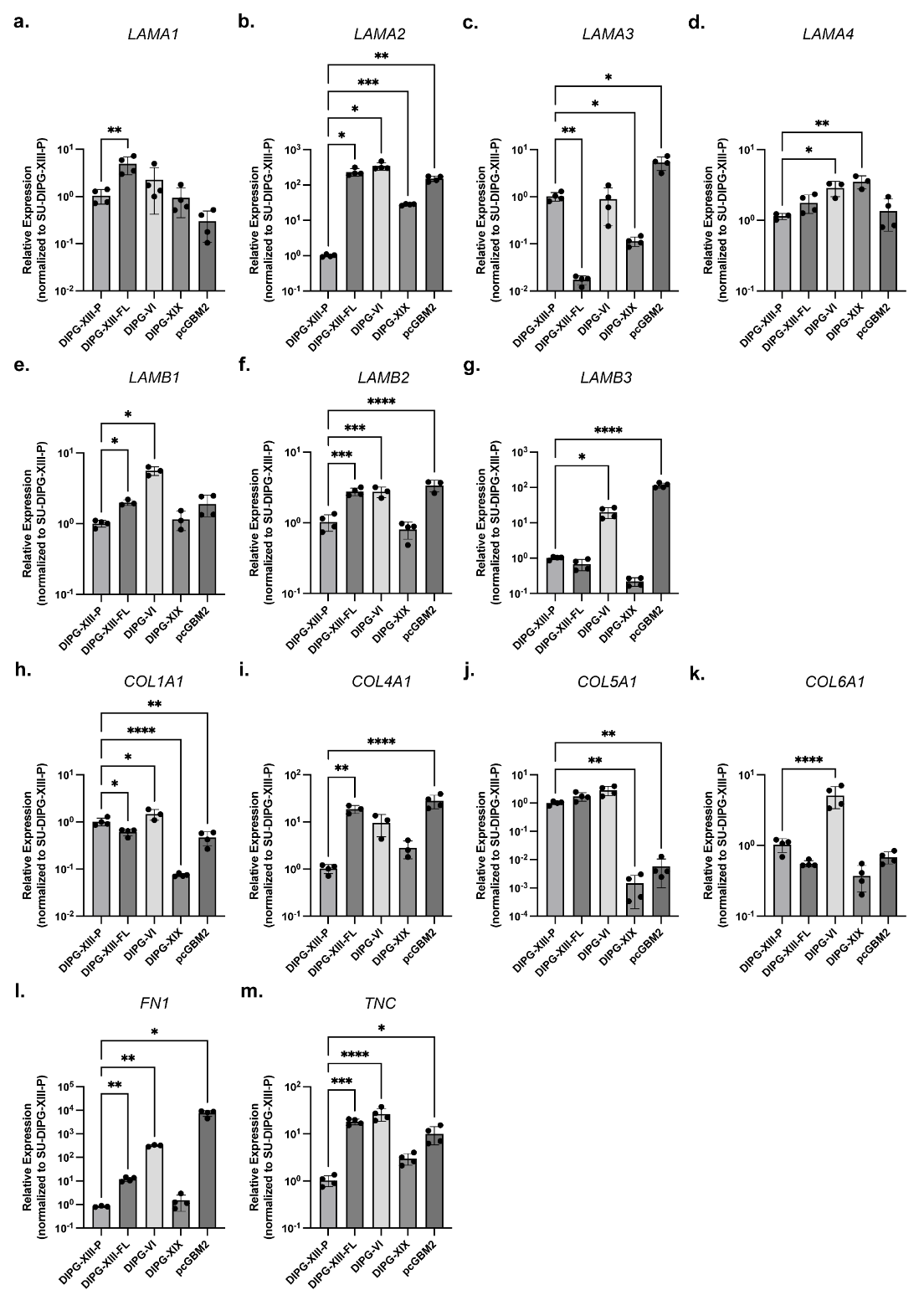
**

**Supplementary Figure 4. Gene expression of ECM components across four patient-derived DIPG cell lines and one pcGBM cell line.** Relative mRNA expression of several ECM components in SU-DIPG-XIII-P, SU-DIPG-XIII-FL, SU-DIPG-VI, SU-DIPG-XIX, and SU-pcGBM2 cell lines. Genes profiled include *LAMA1* **(a)**, *LAMA2* **(b)**, *LAMA3* **(c)**, *LAMA4* **(d)**, *LAMB1* **(e)**, *LAMB2* **(f)**, *LAMB3* **(g)**, *COL1A1* **(h)**, *COL4A1* **(i)**, *COL5A1* **(j)** *COL6A1* **(k)**, *FN1* **(l)**, and *TNC* **(m)**. Expression levels are normalized to GAPDH and SU-DIPG-XIII-P. n ≥ 3 replicates per condition. *p<0.05, **p<0.01, ***p<0.001, ****p<0.0001 by one-way ANOVA with Dunnett’s multiple comparisons test. Data reported in **(a-m)** represent mean value ± s.d.

**
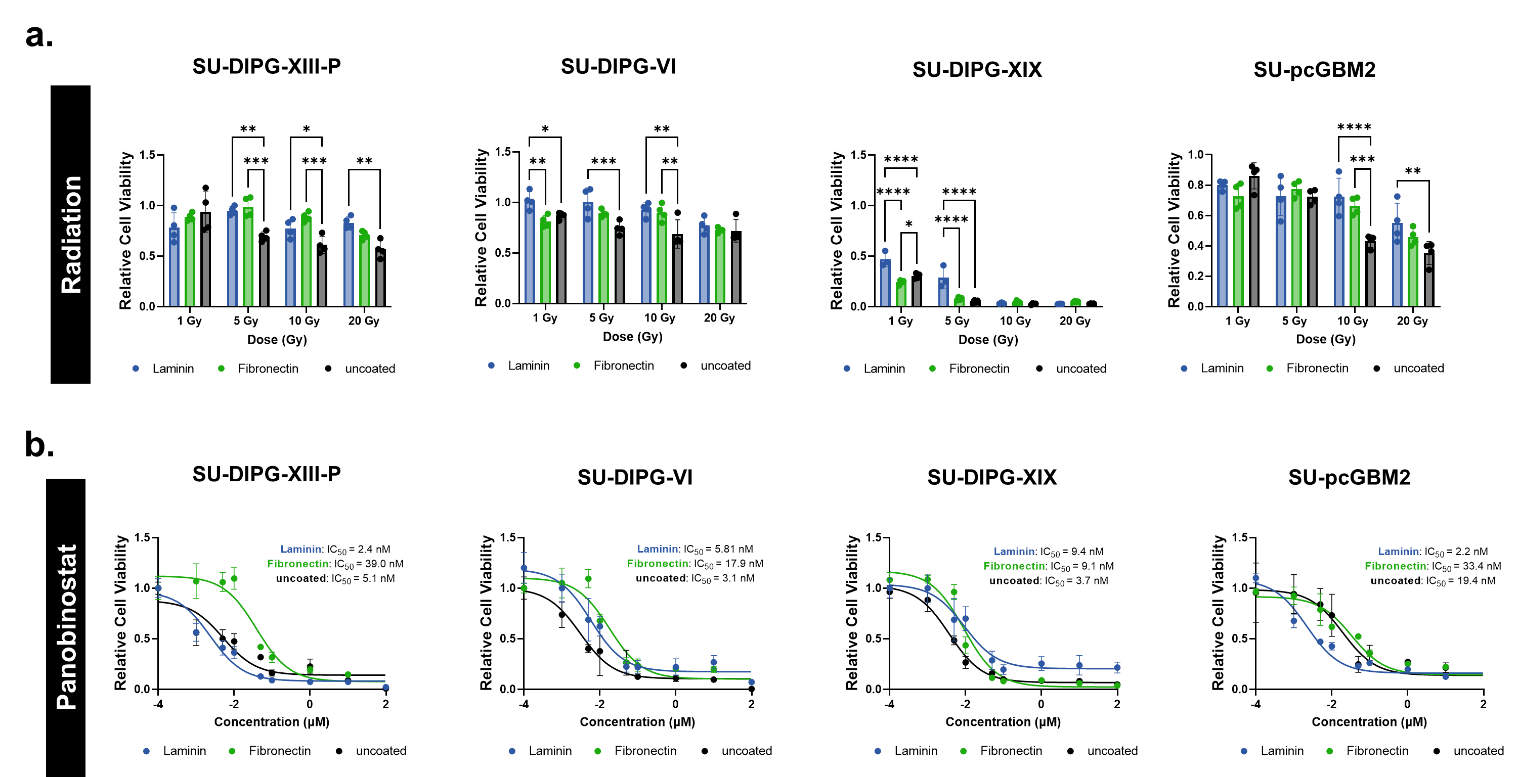
**

**Supplementary Figure 5. The effect of ECM coating on multiple patient-derived pediatric brain cancer cell lines in response to radiation or panobinostat treatment in 2D culture.** Relative cell viability of DIPG or pcGBM cells treated with radiation **(a)** or panobinostat **(b)**. Cells were cultured on 2D tissue culture plastic (TCP) coated with either laminin (50 µg/mL) or fibronectin (50 µg/mL) or on 2D uncoated TCP. n ≥ 4 (radiation) and n = 3 (panobinostat). *p<0.05, **p<0.01, ***p<0.001, ****p<0.0001 by two-way ANOVA with Tukey’s multiple comparisons test. Data reported in **(a)** and **(b)** represent mean value ± s.d.

**
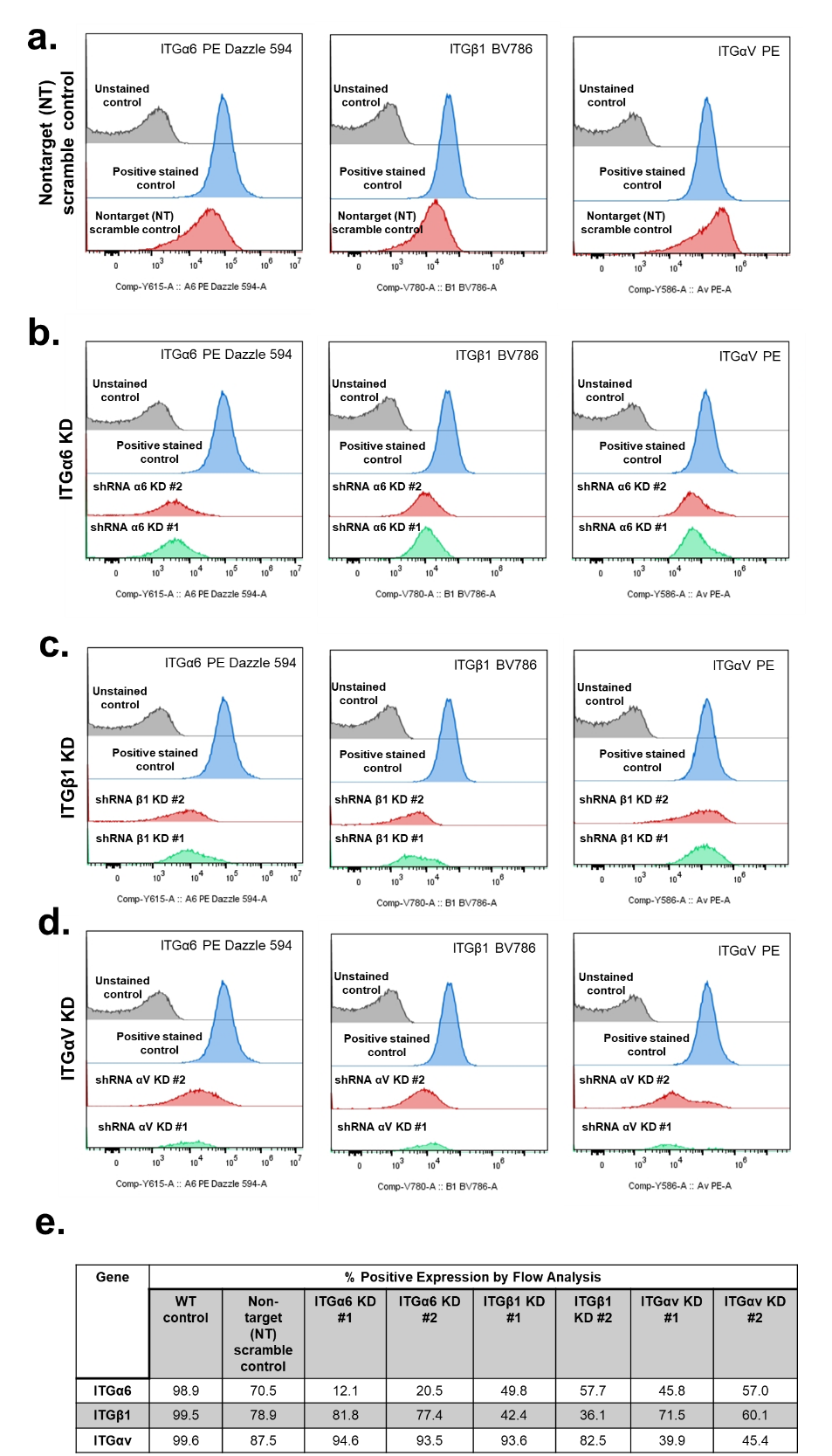
**

**Supplementary Figure 6. Validating integrin knockdown (KD) using flow cytometry. (a-d)** Flow cytometry plots of ITGα6, ITGβ1, and ITGαV expression levels of SU-DIPG-XIII-FL cells treated with shRNA nontarget (NT) scramble control **(a),** shRNA-mediated KD against ITGα6 **(b)**, ITGβ1 **(c)**, or ITGαV **(d)**. **(e)** A table summarizing percentage of ITGα6, ITGβ1, and ITGαV expression in each generated shRNA KD SU-DIPG-XIII-FL cell line.


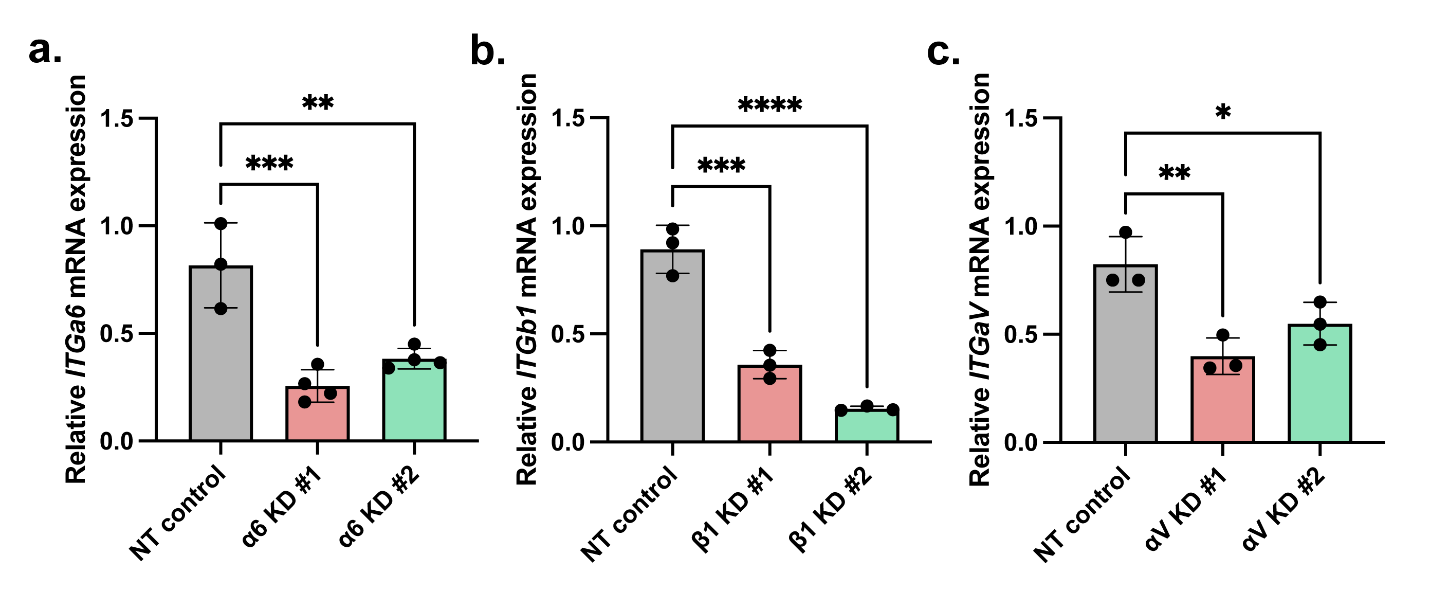


**Supplementary Figure 7. Validating integrin knockdown (KD) using qPCR.** Relative mRNA expression of SU-DIPG-XIII-FL cells treated with shRNA nontarget (NT) scramble control and shRNA-mediated KD against ITGα6 **(a)**, ITGβ1 **(b)**, or ITGαV **(c)**. Expression levels are normalized to GAPDH and WT control. n ≥ 3 replicates per condition. *p<0.05, **p<0.01, ***p<0.001, ****p<0.0001 by one-way ANOVA with Dunnett’s multiple comparisons test. Data reported in **(a-c)** represent mean value ± s.d.

**
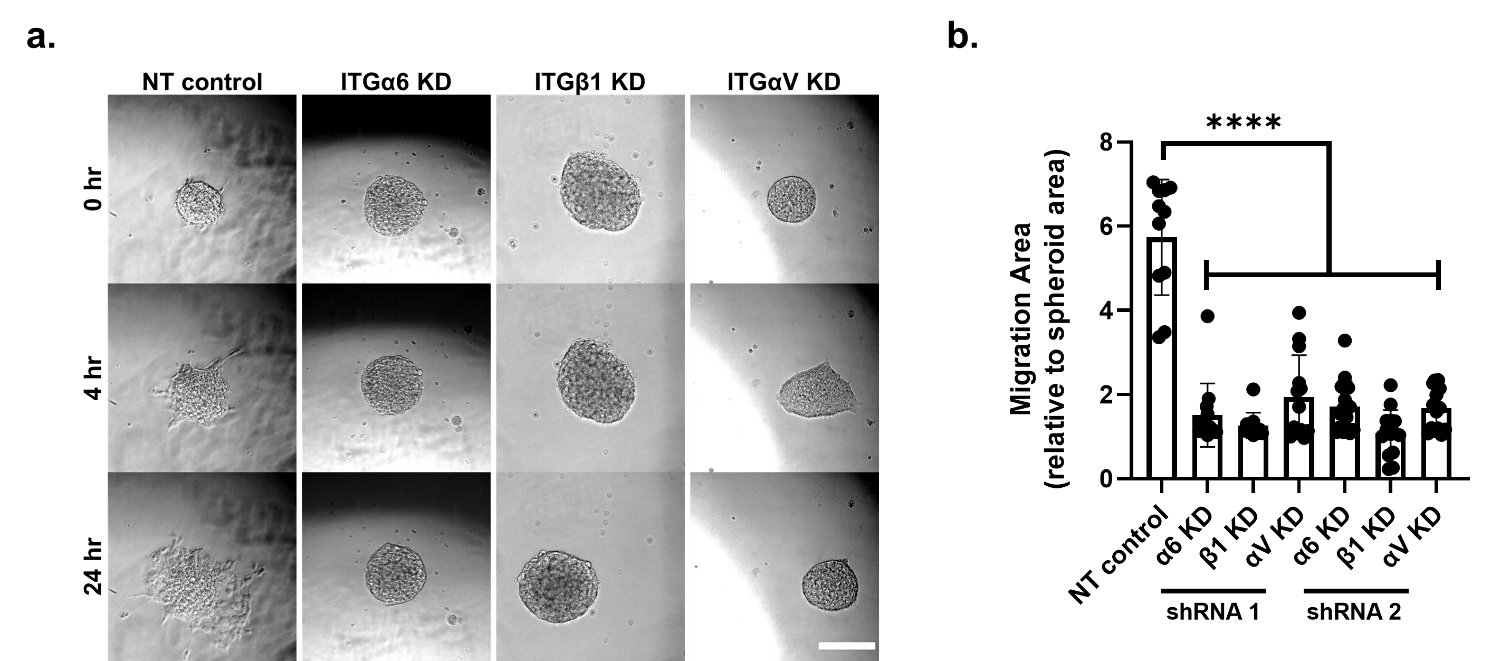
**

**Supplementary Figure 8. shRNA mediated knockdown (KD) of ITGα6, ITGβ1, or ITGαV reduces DIPG migration on 2D. (a)** Brightfield time-lapse imaging of shRNA nontarget (NT) scrambled control DIPG spheroids vs. shRNA integrin KD against ITGα6, ITGβ1, or ITGαV DIPG spheroids cultured on 2D TCP coated with laminin and fibronectin (each at 50 µg/mL). Scale bar, 200 µm. **(b)** Quantification of migration area of DIPG cells. Migration area at 24 hrs is normalized to the spheroid area at 0 hrs. n ≥ 11 DIPG spheroids per group. ****p<0.0001 by one-way ANOVA with Dunnett’s multiple comparisons test. Data reported in **(b)** represent mean value ± s.d.


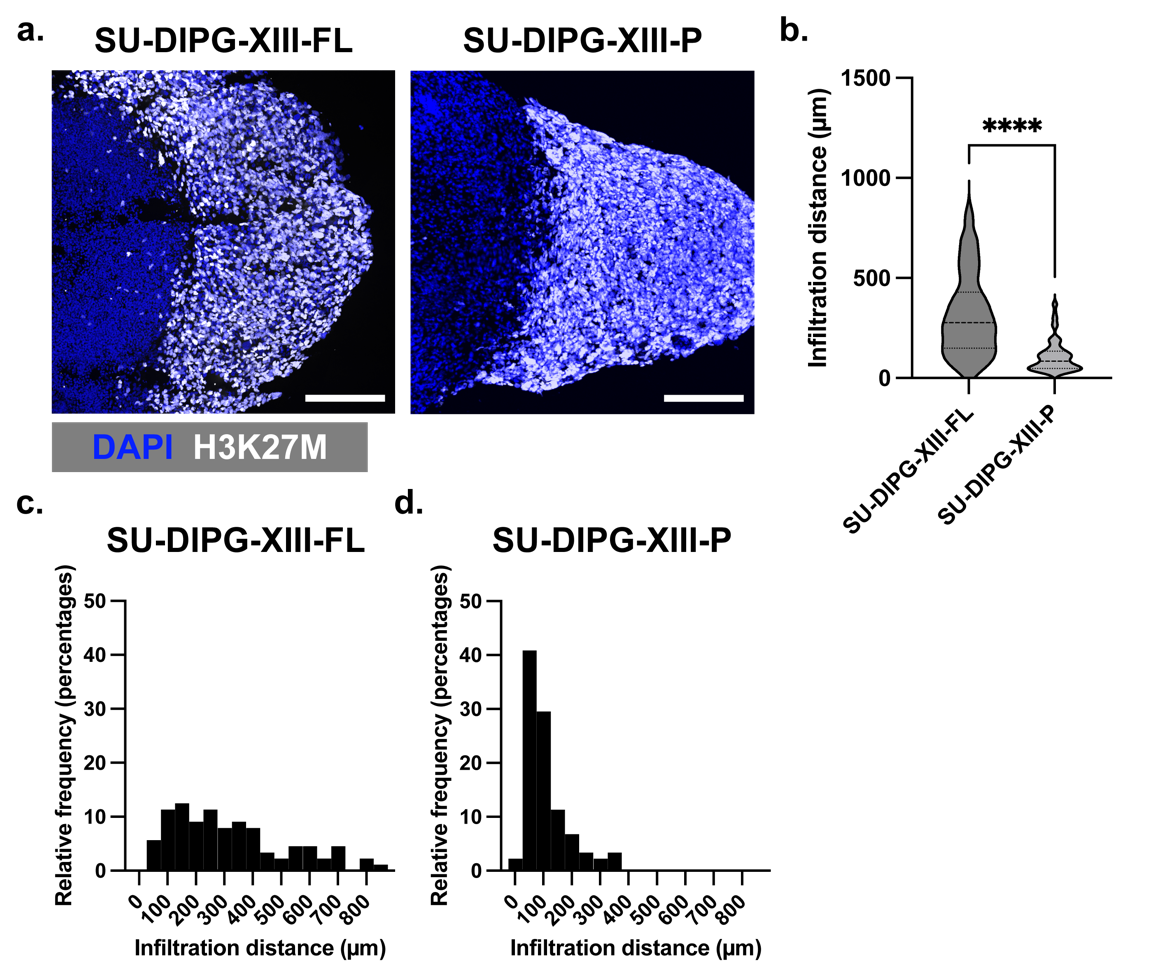


**Supplementary Fig. 9. The metastatic DIPG cell line (SU-DIPG-XIII-FL) exhibits greater infiltration within the DIPG-neural assembloid model than the primary DIPG cell line (SU-DIPG-XIII-P). (a)** Representative immunostaining for Histone H3 K27M (H3K27M, white) and DAPI (blue) at the fusion interface within DIPG-neural assembloids derived from different regions of the same patient. Scale bar, 200 µm. **(b)** Quantification of infiltration distance of H3K27M+ nuclei from the boundary between the DIPG spheroid and the neural organoid (n = 85 cells for SU-DIPG-XIII-FL, n = 88 cells for SU-DIPG-XIII-P). **(c,d)** Histogram of infiltration distances for SU-DIPG-XIII-FL **(c)** and SU-DIPG-XIII-P **(d)**. *p<0.05, **p<0.01, ***p<0.001, ****p<0.0001 by Mann Whitney test.

**
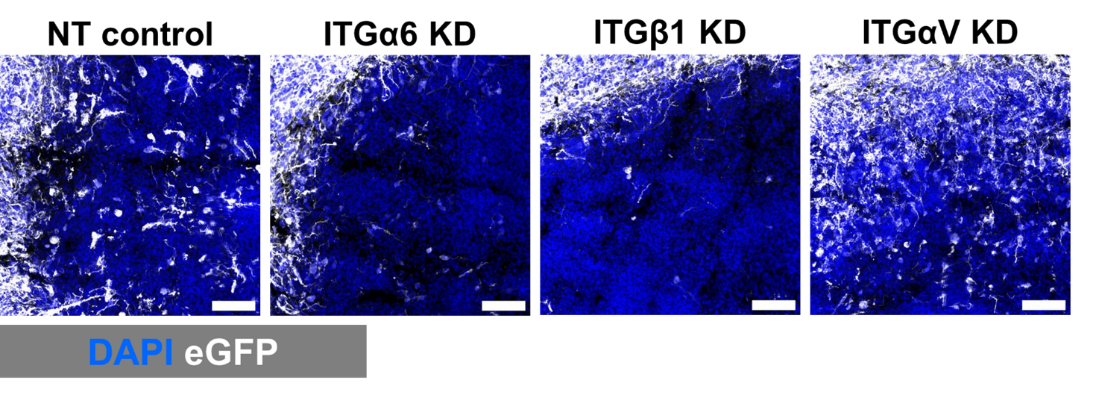
**

**Supplementary Fig. 10. Infiltration of DIPG into neural organoids.** Representative immunostaining images of DIPG infiltration into DIPG-neural assembloids at day 10 of nontarget (NT) scrambled control DIPG spheroids vs. shRNA integrin knockdown (KD) against ITGα6, ITGβ1, or ITGαV DIPG spheroids. DIPG cells are in pseudocolor (white) for eGFP, DAPI (blue). Scale bar, 100 µm.

**
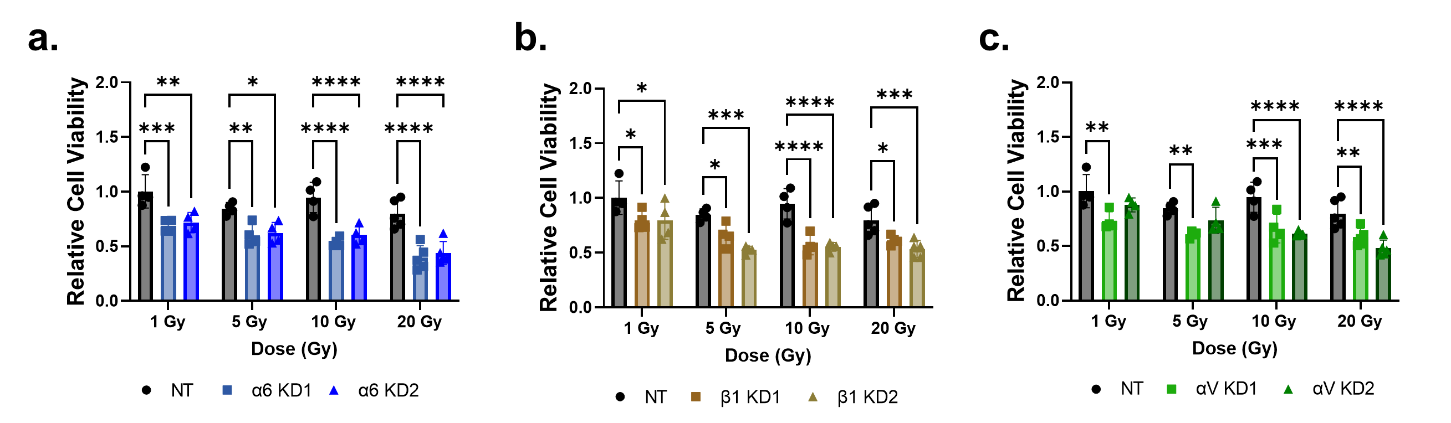
**

**Supplementary Figure 11. The effect of integrin knockdown (KD) on DIPG response to radiation in 2D culture coated with laminin and fibronectin.** Relative cell viability of nontarget (NT) scramble control vs. ITGα6 KD **(a)**, ITGβ1 KD **(b)**, or ITGαV KD **(c)** SU-DIPG-XIII-FL cells when cultured on 2D TCP coated with both laminin and fibronectin. Radiation doses ranged from 1 – 20 Gy. n ≥ 4 replicates per condition. *p<0.05, **p<0.01, ***p<0.001, ****p<0.0001 by two-way ANOVA with Tukey’s multiple comparisons test. Data reported in **(a-c)** represent mean value ± s.d.


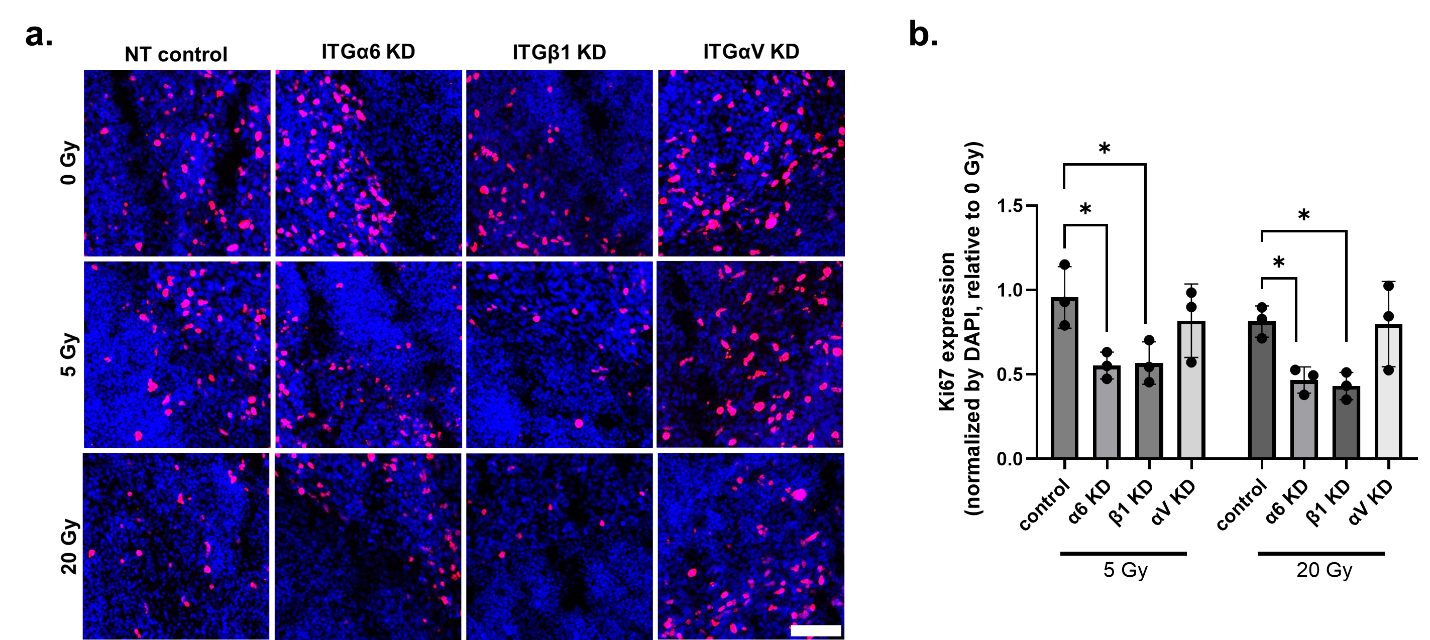


**Supplementary Figure 12. Integrin knockdown of ITGα6 and ITGβ1 reduces proliferation post radiation. (a)** Representative immunostaining for Ki67 (red) and DAPI (blue) at the fusion interface within the DIPG-neural assembloids when irradiated at 0, 5, and 20 Gy. Scale bar, 100 µm. **(b)** Quantification of the relative degree of proliferation based on Ki67 immunostaining at the fusion interface normalized by DAPI and relative to the untreated assembloid control group in each respective KD group. n = 3 assembloids per group. *p<0.05 by one-way ANOVA with Tukey’s multiple comparisons test. Data reported in **(b)** mean value ± s.d.

**
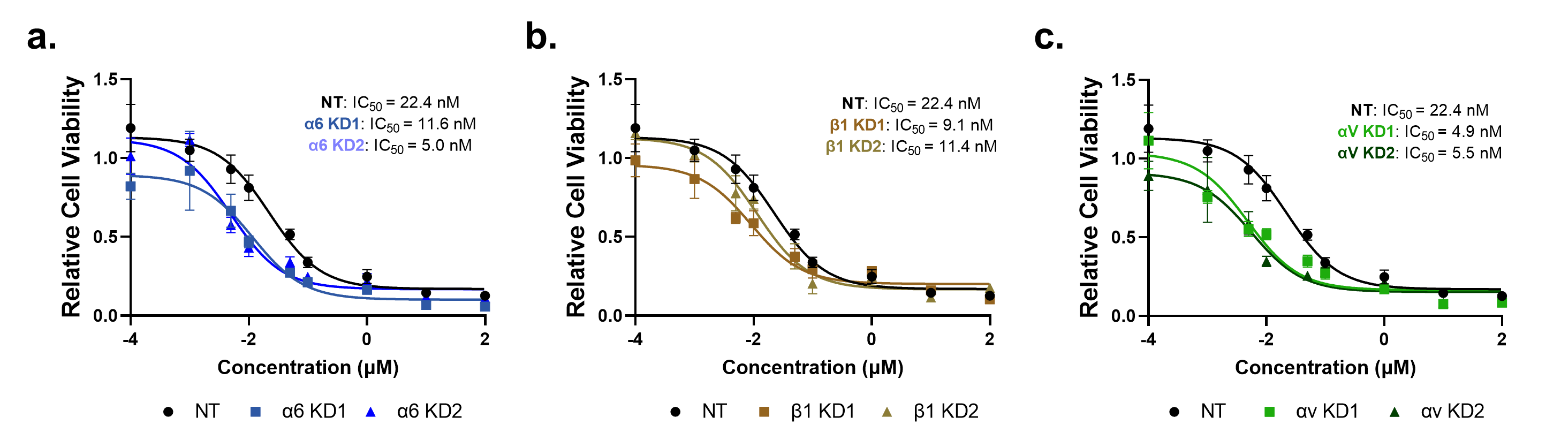
**

**Supplementary Figure 13. The effect of integrin knockdown (KD) on DIPG response to panobinostat in 2D culture coated with laminin and fibronectin.** Relative cell viability of nontarget (NT) scramble control vs. ITGα6 KD **(a)**, ITGβ1 KD **(b)**, or ITGαV KD **(c)** panobinostat treated SU-DIPG-XIII-FL cells when cultured on 2D TCP coated with both laminin and fibronectin. n = 3 replicates per condition. Data reported in **(a-c)** represent mean value ± s.d.

**
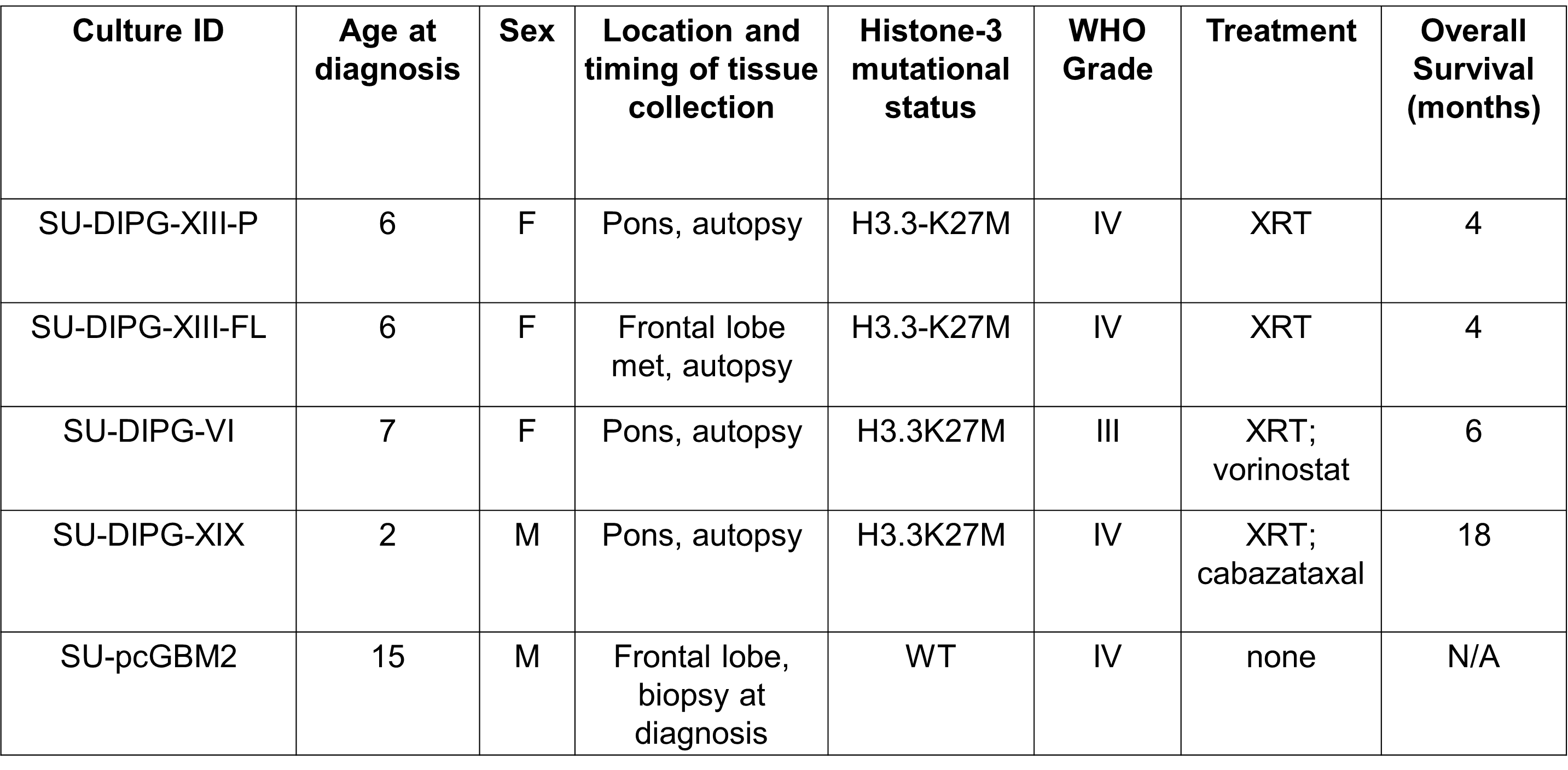
**

**Table S1. Characteristics of patient-derived DIPG and pcGBM cultures.** Legend: DIPG = diffuse intrinsic pontine glioma; pcGBM = pediatric cortical glioblastoma; XRT = radiotherapy; WHO = World Health Organization.

**
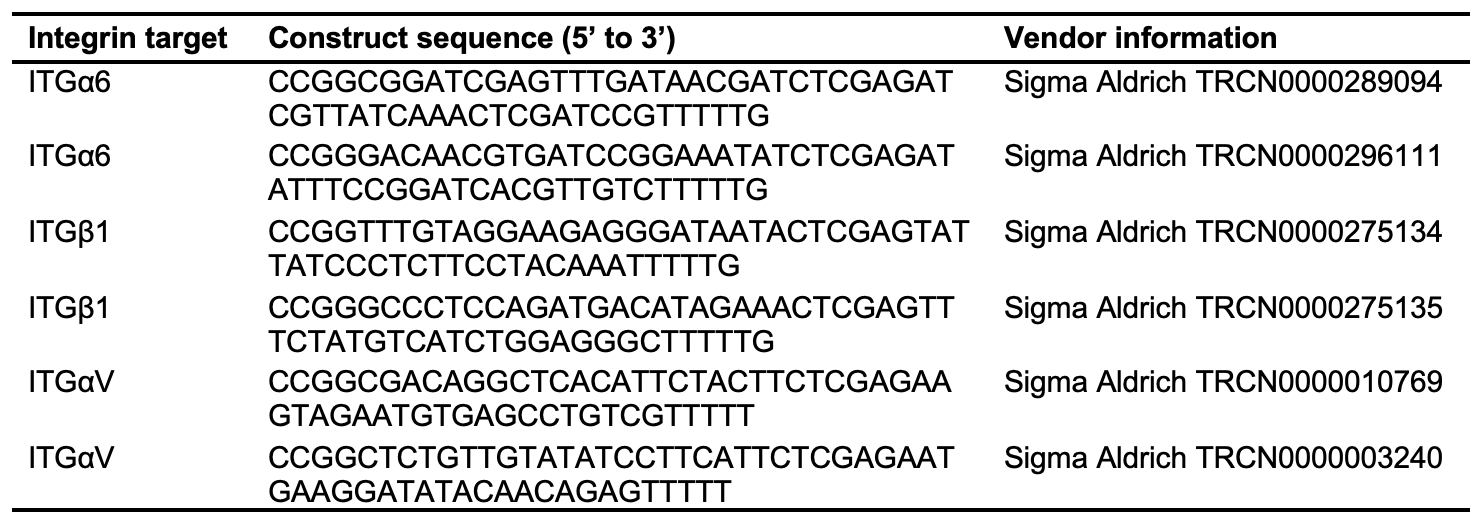
**

**Table S2. shRNA constructs used to generate integrin knockdown DIPG cell lines.**


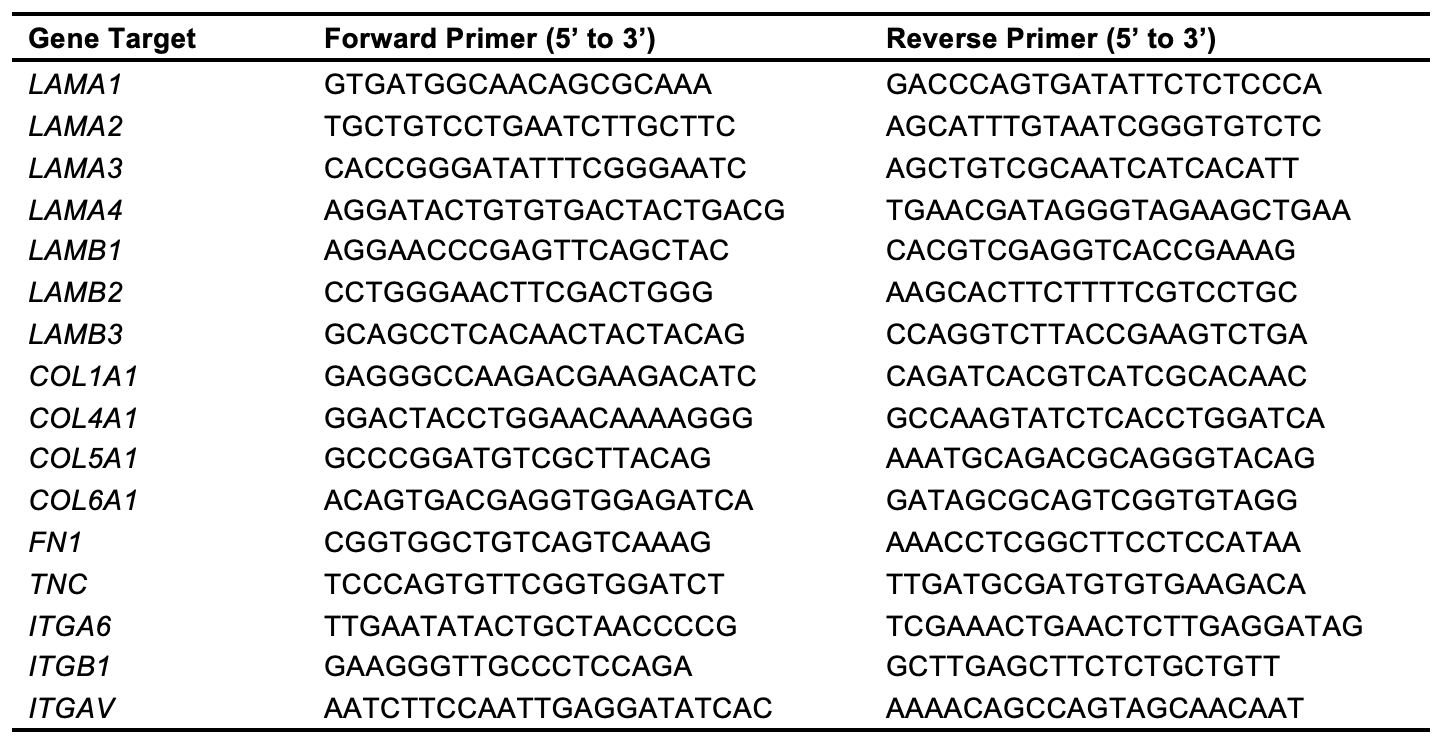


**Table S3. Primers used for qPCR experiments.**

**Table S4. RNA sequencing analyses details and parameters (separate file).** Analysis includes including mean gene expression values, log2(fold-change), p-values, and adjusted p-values (False Discovery Rate).

**Supplementary Videos:**

**Supplementary Video 1:** Representative adhesion and migration of SU-DIPG-XIII-FL spheroid plated on 2D TCP coated with laminin (50 µg/mL). Frames were taken every 20 min over 24 hrs. Scale bar, 200 µm.

**Supplementary Video 2:** Representative adhesion and migration of SU-DIPG-XIII-FL spheroid plated on 2D TCP coated with fibronectin (50 µg/mL). Frames were taken every 20 min over 24 hrs. Scale bar, 200 µm.

**Supplementary Video 3:** Representative adhesion and migration of SU-DIPG-XIII-FL spheroid plated on 2D TCP coated with collagen-IV (50 µg/mL). Frames were taken every 20 min over 24 hrs. Scale bar, 200 µm.

**Supplementary Video 4:** Representative adhesion and migration of SU-DIPG-XIII-FL spheroid plated on 2D TCP coated with collagen-I (50 µg/mL). Frames were taken every 20 min over 24 hrs. Scale bar, 200 µm.

**Supplementary Video 5:** Representative adhesion and migration of non-target, scrambled shRNA control SU-DIPG-XIII-FL spheroid plated on 2D TCP coated with laminin and fibronectin (each at 50 µg/mL). Frames were taken every 10 min over 24 hrs. Scale bar, 200 µm.

**Supplementary Video 6:** Representative adhesion and migration shRNA mediated ITGα6 knockdown of SU-DIPG-XIII-FL spheroid plated on 2D TCP coated with laminin and fibronectin (each at 50 µg/mL). Frames were taken every 10 min over 24 hrs. Scale bar, 200 µm.

**Supplementary Video 7:** Representative adhesion and migration shRNA mediated ITGβ1 knockdown of SU-DIPG-XIII-FL spheroid plated on 2D TCP coated with laminin and fibronectin (each at 50 µg/mL). Frames were taken every 10 min over 24 hrs. Scale bar, 200 µm.

**Supplementary Video 8:** Representative adhesion and migration shRNA mediated ITGαV knockdown of SU-DIPG-XIII-FL spheroid plated on 2D TCP coated with laminin and fibronectin (each at 50 µg/mL). Frames were taken every 10 min over 24 hrs. Scale bar, 200 µm.
